# Striatal ensembles specify and control granular forelimb actions

**DOI:** 10.64898/2025.12.03.692128

**Published:** 2025-12-08

**Authors:** Ines Rodrigues-Vaz, Vivek R. Athalye, Darcy S. Peterka, Rui M. Costa

**Affiliations:** 1 Zuckerman Mind Brain Behavior Institute, Departments of Neuroscience and Neurology; Columbia University; New York, NY, 10027; USA; 2 Champalimaud Neuroscience Programme, Champalimaud Centre for the Unknown, Lisbon 1400-038, Portugal; 3 Allen Institute, Allen Institute for Neural Dynamics, Seattle, WA, 98109; USA; 4 Aligning Science Across Parkinson’s (ASAP) Collaborative Research Network, Chevy Chase, MD20815; 5 Kavli Institute for Brain Science, Columbia University; New York, NY 10027; USA

## Abstract

The ability of the brain to control specific fine actions is crucial for survival. The striatum is a critical brain center for both movement and learning, and its dysfunction underlies numerous movement disorders^1–9^. Whereas activity in the striatum has been classically viewed as invigorating^5,10–13^ and reinforcing movements^12–20^, recent studies suggest that striatal activity encodes specific movements^21–24^. However, it is not known how granular this activity is, and if it indeed controls specific ongoing movements. We designed a task where mice performed two minimally-different forelimb actions, consisting of a push or pull isometric force on an immobile joystick, and imaged the activity of medium spiny neurons (MSNs) in the dorsolateral striatum using 2-photon microscopy. We observed that striatal activity encoded both the preparation and execution of specific actions, even when those actions were not reinforced. Furthermore, both populations of D1 and D2-MSNs - classically viewed as promoting versus inhibiting movement^2,3,25^ - equally encoded action identity. We developed a closed-loop system to model and stimulate action-specific neural ensembles deep in the brain, using holographic optogenetics through a GRIN lens. Stimulation of action-specific ensembles of both D1- and D2-MSNs increased the force of ongoing actions, but only when the ensemble stimulated was congruent with the ongoing action. These results reveal that specific ensembles of both D1- and D2-MSNs causally control specific ongoing actions, as granular as different muscle co-contractions of the same forelimb. Such granularity provides a mechanistic framework for understanding how striatal dysfunction can produce highly specific movement impairments in Huntington’s disease^2,9^ and dystonia^2,6^.

The brain must generate signals that execute appropriate actions to achieve desired outcomes, and many behaviors demand granular motor control. Writing, for example, requires precisely coordinated muscle co-contractions in the arm to apply specific forces to a pen. While motor cortices, brainstem, and spinal circuits are classically linked^26^ to the online specification and execution of movement kinematics and force, the striatum has been primarily framed as a center for invigoration^5,10–13^ and reinforcement^12–20^ rather than as a controller of ongoing action per se. This view has been supported by the fact that Parkinson’s disease, where dopamine inputs to striatum are depleted, leads to deficits in action initiation^4^, vigor^5^, and reinforcement^7,8^. Alternatively, accumulating evidence suggests that striatal activity can encode specific actions^21–24^, raising the possibility that the striatum contributes to action specification rather than merely broadcasting a more generic “go/no-go”^27^ or vigor signal. This is supported by observations that in disorders where striatal neurons are affected, like in Huntington’s disease^9^ or dystonia^2,6^, the execution of particular specific movements is disrupted. Therefore, it is important to determine if the striatum can encode specific ongoing actions, and especially how granular this encoding is. For example, the encoding in striatum may simply reflect actions performed by different body parts, as suggested by its topography, or at the other extreme resolve even subtle differences in co-contraction patterns within the same limb. Furthermore, it is critical to determine whether this encoding is merely corollary or if it indeed controls specific ongoing actions moment-to-moment.

To address this, we developed a motor task in which head-fixed mice executed two minimally different isometric forelimb actions, consisting of push or pull forces on an immobile joystick, performed from a constrained posture without overt limb movement. We designed it so that both actions rely on similar forelimb muscle co-contractions, differing only subtly in pattern and direction of force. This allowed us to then use 2-photon imaging to test whether activity of medium spiny neurons (MSNs) encodes and predicts the identity of ongoing actions at a fine, granular level. Furthermore, it allowed us to test whether action specification can happen independently of reinforcement, and also whether preparation and execution are differently encoded.

Although D1- and D2-MSNs have been canonically assigned opponent roles in movement promotion and suppression^2,3,25,28^, both receive cortical inputs that carry specific movement plans^29–33^, both are activated at movement initiation^34,35^, and both seem necessary for execution^35,36^. We therefore tested whether D1- and D2-MSNs would encode the identity of specific isometric actions differently or similarly.

Finally, to establish if specific striatal activity can causally control specific ongoing action, we combined deep two-photon imaging through a GRIN lens with holographic optogenetics^37–39^ in a closed-loop paradigm. This enabled us to model the population dimensions that predict specific actions and forces, and identify small, action-specific ensembles within spatially intermingled D1- and D2-MSNs, and then stimulate those ensembles precisely during self-paced actions with millisecond latency. We thus tested whether stimulating action-specific ensembles increases the force of the ongoing action, and whether this specificity holds for both D1- and D2-MSNs.

Together, this strategy allowed us to determine that specific ensembles of striatal neurons control granular ongoing actions of the forelimb that are as fine as distinct patterns of muscle co-contraction of extensor and flexor muscles. Further, striatal ensembles of both D1- and D2-MSNs specify action identity, independently of whether or not the action is reinforced.

## Mice learn to execute specific isometric forelimb actions

**Fig 1:**
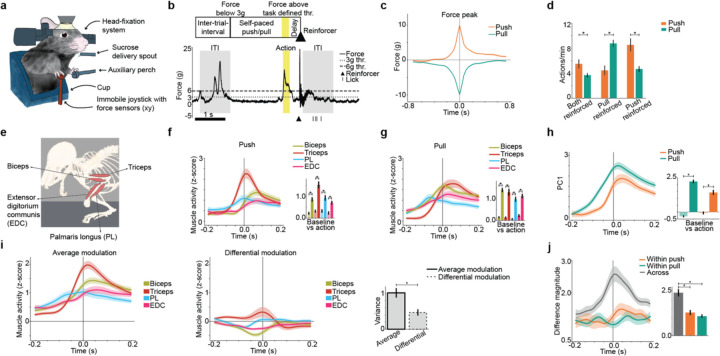
Mice execute two isometric forelimb actions using similar muscle patterns. **a,** Task schematic. **b,** Task events for reinforcement of self-paced actions. Trace of force applied to the joystick on an example trial. **c**, Trial-averaged force trace of 6g cross actions time locked to force peak. By our convention, force in the push direction is positive and pull direction is negative. Data are mean+s.e.m. across 109 sessions from 8 mice. **d,** Mice performed both actions and increased the action which led to sucrose reinforcement. Data are mean+s.e.m. from 8 mice, averaging over sessions in each block (Methods). Mice performed push more than pull when both actions were reinforced (see Extended Data Table 1, table ref. 1.1). Mice performed pull more than push when pull was reinforced (see Extended Data Table 1, table ref. 1.2). Mice performed push more than pull when push was reinforced (see Extended Data Table 1, table ref. 1.3). **e,** Schematic of forelimb muscles simultaneously recorded with EMG. **f,** Trial-averaged z-scored EMG of 6g cross push actions locked to 6g cross, force-matched with pull actions (Methods). Each muscle significantly activated during action (see Extended Data Table 1, table ref. 1.4–1.7). **g,** Same as “f” but for pull action. Each muscle significantly activated during action (see Extended Data Table 1, table ref. 1.8–1.11). **h,** Projection of EMG onto the first principal component (PC1). PC1 increased significantly for both actions (see Extended Data Table 1, table ref. 1.12,1.13). **i,** Average and differential EMG modulation across actions. Right: variance of modulation. Variance of average modulation was greater than differential modulation (see Extended Data Table 1, table ref. 1.14). **j,** Vector magnitude of EMG difference across actions versus within an action. The difference across actions is greater than within each action (see Extended Data Table 1, table ref. 1.15,1.16). **f-j,** Data are mean+s.e.m. over n=41 sessions from 4 mice.

To investigate whether the striatum encodes and controls minimal and specific actions of the same body part, we developed a task in which mice performed two forelimb actions (Fig. 1a), consisting of a push or pull force on an immobile joystick in the absence of overt movement (i.e. isometric co-contractions, see Methods). Mice initiated actions in a self-paced manner, in the absence of external cues. We defined two targets as push and pull that exceed thresholds of force and duration determined for each session (Fig. 1bc, Methods), and these actions triggered reinforcement delivered as a delayed sucrose reward (5 ul per reward). We trained mice in blocks, where reinforcement was triggered for both actions or just one action (Methods). Mice generally had a bias for push when both actions were reinforced. Importantly they were able to learn which specific action was reinforced when only one was reinforced, increasing the rate of pulls when pull was reinforced, and the rate of pushes when push was reinforced (Fig. 1d).

We characterized the simultaneous activity of four forelimb muscles - biceps, triceps, palmaris longus (PL), and the extensor digitorium communis (EDC) - during the performance of these isometric actions (Fig. 1e). Both isometric push and pull were executed with co-contraction of extensors and flexors, i.e by activating antagonist muscle pairs to different extents (Fig. 1f,g). The dominant dimension of muscle activity increased for both actions (Fig. 1h), and the sum of muscle activity across actions showed more variance than the difference between actions (Fig. 1i). While the actions were similar, forelimb muscles showed significant differential activation (Fig. 1jk). Thus, mice executed actions composed of similar yet specific muscle patterns.

## Striatal ensembles encode isometric actions

**Fig 2:**
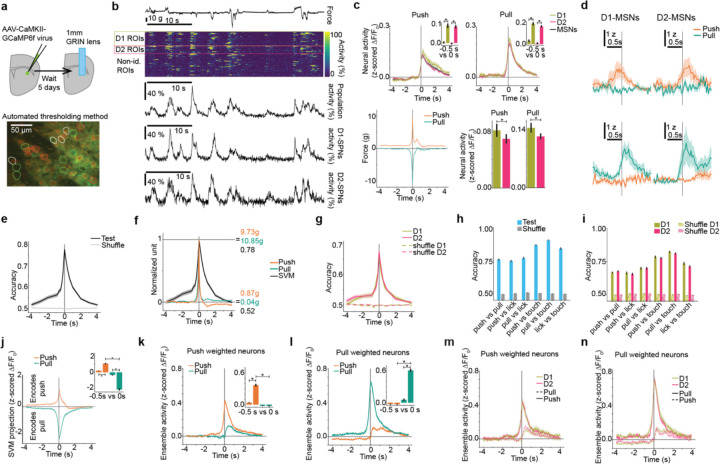
D1- and D2-MSNs both encode action identity. **a,** Schematic of experimental procedures; Top: GCaMP6f virus injection and lens implant in transgenic mice to image D1- and D2-MSNs simultaneously; Bottom: Schematic of automated method for identifying D1- and D2-MSNs based on transgenic expression of tdTomato (Methods). **b,** Example trace of force, individual neuronal activity for all ROIs in this field of view, population activity averaged across neurons, averaged activity for D1-MSNs (n=21 neurons) and averaged activity for D2-MSNs (n=21 neurons). Neural activity is ΔF/F_0_**. c,** Population activity (top) and force (bottom left) locked to peak force of push (top left) and pull (top right) actions, averaged across D1-, D2-, and all MSNs. Data are mean+s.e.m. across 86 sessions from 8 mice. D1-MSNs increased their neural activity at push force peak (“0 s”) relative to “−0.5 s” (see Extended Data Table 1, table ref. 2.1) D2 -MSNs increased their neural activity at push force peak (“0 s”) relative to “−0.5 s” (average 3 frames around each time) (see Extended Data Table 1, table ref. 2.2) ; D1-MSNs increased their neural activity at pull force peak (“0 s”) relative to “−0.5 s” (see Extended Data Table 1, table ref. 2.3); D2-MSNs increased their neural activity at pull force peak (“0 s”) relative to “−0.5 s” (average 3 frames around each time) (see Extended Data Table 1, table ref. 2.4) ; D1-MSNs are more active than D2-MSNs around force peak (see Extended Data Table 1, table ref. 2.5,2.6); **d,** Example traces of individual D1- or D2-MSN activity locked to push and pull actions. **e,** Decoding MSN activity to predict action identity on single trials with a model fit at each time lag relative to force peak. Accuracy at force peak was greater than a model trained with shuffled action identity: “shuffle” (see Extended Data Table 1, table ref. 2.7). Accuracy was greater than shuffle when averaged over time points in the 4 seconds before force peak (see Extended Data Table 1, table ref. 2.8)and in the 0.5 seconds before force peak during which there was no other action (see Extended Data Table 1, table ref. 2.9). Data are mean+s.e.m. across n=87 sessions from 8 mice. **f,** Decoding MSN activity overlayed on the average force of actions (normalized to −4s and peak at 0 s). **g,** Decoding D1- or D2- MSN activity. Accuracy at force peak was greater than shuffle for D1-MSNs (see Extended Data Table 1, table ref. 2.10) and D2-MSNs (see Extended Data Table 1, table ref. 2.11), with no significant difference between D1- and D2- MSNs (see Extended Data Table 1, table ref. 2.12). Accuracy was greater than shuffle when averaged over time points in the 4 seconds before force peak for D1-MSNs (see Extended Data Table 1, table ref. 2.13) and D2-MSNs (see Extended Data Table 1, table ref. 2.14) and in the 0.5 seconds before force peak for D1-MSNs (see Extended Data Table 1, table ref. 2.15) and D2-MSNs (see Extended Data Table 1, table ref. 2.16). **h,** Decoding MSN activity at force peak for push and pull, predicting action identity between pairs of actions including push, pull, touch, and licking. Accuracy was greater than shuffle for all action pairs (see Extended Data Table 1, table ref. 2.17–2.22). **i,** Same as g, decoding D1- and D2-MSNs activity. Decoding push versus pull was not significantly different between D1- and D2-MSNs (see Extended Data Table 1, table ref. 2.23). Accuracy was greater than shuffle for all action pairs for D1-MSNs (see Extended Data Table 1, table ref. 2.24–2.29) and D2-MSNs (see Extended Data Table 1, table ref. 2.30–2.35). **j-l,** Data are mean+s.e.m. across 86 sessions from 8 mice. **j,** Projection of population activity (z-scored ΔF/F_0_) on the SVM dimension locked to force peak. Inset: average SVM projection in the 500 ms before force peak (“−0.5 s”) and at force peak (“0 s”). For the push action, the SVM projection at force peak (“0 s”) increased relative to “−0.5 s” (see Extended Data Table 1, table ref. 2.36). For the pull action, the SVM projection at force peak (“0 s”) decreased relative to “−0.5 s” (see Extended Data Table 1, table ref. 2.37). The SVM projection at force peak was greater for push than for pull (see Extended Data Table 1, table ref. 2.38). **k,** Ensemble activity (z-scored ΔF/F_0_) averaged across push weighted neurons, locked to force peak for push and pull actions. Inset: average activity at −0.5 s before force peak and at force peak for each action. The activity of push weighted neurons increased at push force peak (“0 s”) relative to “−0.5 s” (see Extended Data Table 1, table ref. 2.39). Activity at push force peak was greater than at pull force peak (see Extended Data Table 1, table ref. 2.40). **l,** Ensemble activity (z-scored ΔF/F_0_) averaged across pull weighted neurons, locked to force peak for push and pull actions. Inset: average activity at −0.5 s before force peak and at force peak for each action. The activity of pull weighted neurons increased at pull force peak (“0 s”) relative to “−0.5 s” (see Extended Data Table 1, table ref. 2.41). Activity at pull force peak was greater than at push force peak (see Extended Data Table 1, table ref. 2.42). **m,** Same as Fig. 2k for D1- and D2-MSNs. Activity of push weighted D1- and D2-MSNs around push force peak (-0.5 s to 0.5 s) is not significantly different (see Extended Data Table 1, table ref. 2.43); Data are mean+s.e.m. across 63 sessions from 8 mice. **n,** Same as Fig. 2l for D1- and D2-MSNs. Activity of pull weighted D1- and D2-MSNs around pull force peak (-0.5 s to 0.5 s) is not significantly different (see Extended Data Table 1, table ref. 2.44); Data are mean+s.e.m. across 73 sessions from 8 mice.

To test whether the striatum encodes the identity of minimal forelimb actions, we measured the simultaneous activity of D1- and D2-MSNs in the forelimb region of dorsolateral striatum (DLS) (Fig. 2a,b). To identify D1- and D2-MSNs within the same subject^40^, we used transgenic mice that expressed the red label tdTomato either in D1-MSNs (T6 and Ai9xEY217) or D2-MSNs (Ai9xAdora2cre), and we developed an automated method to classify red versus non-red neurons (Extended Data Fig.1). We virally expressed GCaMP6f pan-neuronally in DLS and used 2-photon microscopy through a 1mm diameter GRIN lens to image neuronal calcium activity as a proxy for action potentials. We were able to image up to ~200 neurons simultaneously, and the fluorescence signal in both color channels was stable across months of animal training (Extended Data Fig.6 a,b). We identified ongoing isometric actions that exceeded 3g of force for ~66 ms duration (two imaging frames) and that were temporally isolated (i.e. had no preceding action in the 0.5 seconds before onset) (Fig. 2c, bottom-left). The activity of D1- and D2-MSNs was modulated for both actions (Fig. 2b,c), with slightly greater activity in D1-MSNs.

Many individual neurons modulated their activity only during a specific action (Fig. 2d). We therefore asked whether the spatiotemporal activity of MSNs encoded the identity of ongoing actions. We extracted the vector of MSN population activity averaged in 150 ms bins (5 imaging frames) centered at time lags relative to force peak for each trial, and we decoded single-trial population activity into action identity (push or pull) using a linear support vector machine (training in 90% of data, testing in 10% held out data). For time lags before (after) force peak, we analyzed trials with no other action in the 0.5 seconds before (after) force peak. Population activity predicted action identity well above chance with peak accuracy at the time of peak force (Fig. 2ef). In addition, population activity predicted action identity in advance of force peak (Fig. 2f). Interestingly, both D1- and D2-MSNs encoded action identity with equal accuracy and timing (Fig. 2g).

Population activity at force peak predicted action identity even for trials when other actions were allowed in the 0.5s temporal window before and after (Fig. 2h), and across additional actions (Extended Data Fig. 2a–e) such as joystick touch and lick bouts. D1- and D2-MSNs encoded these actions with equal accuracy (Fig. 2i).

Within the high dimensional space of population activity where each neuron’s instantaneous activity is one axis, the SVM identifies a single dimension that best predicts the animal’s action at the time of the force peak. It follows that the projection of population activity on that SVM dimension (a weighted sum over neurons) showed differential modulation at force peak for push and pull actions (Fig. 2j). The SVM’s weights partition neurons into action-specific groups whose activation biases the decoder to predict push or pull. The push-weighted neurons increased activity at push force peak, but not for pull force peak (Fig. 2k). Similarly, the pull weighted neurons increased activity at pull force peak but not for push force peak (Fig. 2l). D1- and D2-MSNs showed no difference in SVM weights (Extended Data Fig. 2f) and action-specific modulation of push- and pull-weighted neurons (Fig. 2m,n). Together, these results demonstrate that striatal population activity robustly encodes the identity of ongoing forelimb actions, with equal contributions from D1- and D2-MSNs.

## Striatal ensembles encode preparation independently of execution

Motor cortices are thought to prepare movement using dimensions of population activity that are different than movement execution^29,30,33^, whereas the basal ganglia are thought to transmit an “On/Off” signal to trigger execution^27,41,42^ or a signal that invigorates execution. We therefore asked whether the striatum distinctly encodes preparation and execution of self-paced actions.

**Fig 3:**
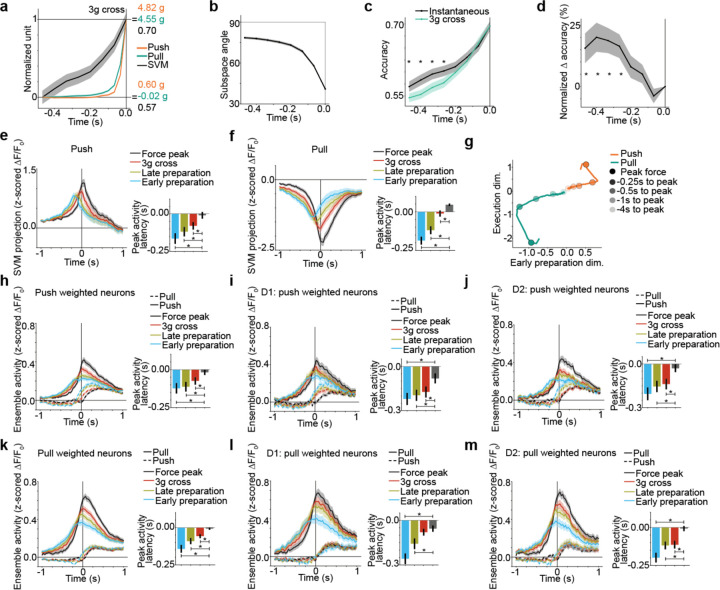
D1- and D2-MSN ensembles progressively encode action preparation distinctly from execution. **a,** Decoding of action identity in the 0.5 seconds before force crosses 3g, during which there are no other actions. The force trace of actions is overlaid. Accuracy was greater than shuffle when averaged over time points in the 0.5 seconds before and including 3g cross (see Extended Data Table 1, table ref. 3.1). Decoding increased earlier than force, as normalized decoding accuracy was larger than normalized force in the 0.5 seconds before 3g cross for the push and pull actions (see Extended Data Table 1, table ref. 3.2, 3.3) . **b,** Alignment of the decoder fit at each time point before 3g cross to the decoder optimized at force peak, as measured with the angle between the decoder weight vectors. Angle decreased with time point for decoder fitting (see Extended Data Table 1, table ref. 3.4). The angle between the force peak decoder and 3g cross decoder was significantly lower than the angle between the force peak decoder and decoders from time points in the 0.5 seconds preceding 3g cross (see Extended Data Table 1, table ref. 3.5–3.11); **c,** Accuracy of decoders optimized at each time point before 3g cross (“Instantaneous”) to the decoder optimized at 3g cross. There was a significant difference between decoders averaging timepoints in the 0.5 seconds before 3g cross (see Extended Data Table 1, table ref. 3.12). Individual timepoints with significant differences are marked with asterisks (see Extended Data Table 1, table ref. 3.13–3.16). **d,** The difference in accuracy between decoders shown in c, normalized by the range of 3g cross decoder accuracy in the 0.5 second time window. **e,** Population activity of push action projected onto the SVM dimension, time locked to force peak. SVMs were optimized for different time points relative to force peak and 3g cross. Inset: Time of peak SVM projection relative to force peak. Peak projection (99^th^ percentile) for early preparation, late preparation, and 3g cross SVMs occurred significantly before the peak force SVM: 3g cross, late prep. and early prep. SVM peaked before peak force SVM (see Extended Data Table 1, table ref. 3.17, 3.18, 3.19). The time of peak SVM projection increased progressively across early preparation, late preparation, 3g cross, and force peak SVMs (see Extended Data Table 1, table ref. 3.20). **f,** Population activity of pull action projected onto the SVM dimension. SVMs were optimized for different time points relative to force peak and 3g cross. Inset: Time of peak SVM projection relative to force peak. Peak projection (99^th^ percentile) for early preparation, late preparation, and 3g cross SVMs occurred significantly before the peak force SVM: 3g cross, late prep. and early prep. SVM peaked before peak force (see Extended Data Table 1, table ref. 3.21, 3.22, 3.23). The time of peak SVM projection increased progressively across early preparation, late preparation, 3g cross, and force peak SVMs (see Extended Data Table 1, table ref. 3.24). **a-f,** Data are mean+s.e.m. across 86 sessions from 8 mice. **g,** Trajectory of population activity for push and pull actions projected onto the peak force SVM (“Execution dim.”, black average data from Fig. 3ef) and early preparation SVM (“Early preparation dim.”, blue average data from Fig. 3ef). Data are mean across 86 sessions from 8 mice. **h,** Ensemble activity averaged across push weighted neurons from each SVM (same SVMs as Fig. 3ef). Inset: Time of peak neural activity relative to force peak. For the push action, peak ensemble activity (90^th^ percentile) from early preparation, late preparation, and 3g cross SVMs occurred significantly before the peak force SVM: 3g cross, late prep. and early prep. SVM peaked before peak force (see Extended Data Table 1, table ref. 3.25, 3.26, 3.27). The time of peak ensemble activity increased progressively across early preparation, late preparation, 3g cross, and force peak SVMs (see Extended Data Table 1, table ref. 3.28). Data are mean+s.e.m. across 86 sessions from 8 mice. **i,** Same as Fig. 3h for push weighted D1-MSNs. For the push action, 3g cross, late prep. and early prep. SVM peaked before peak force (see Extended Data Table 1, table ref. 3.29, 3.30, 3.31).The time of peak ensemble activity increased progressively across early preparation, late preparation, 3g cross, and force peak SVMs (see Extended Data Table 1, table ref. 3.32). Data are mean+s.e.m. from 67 sessions across 8 mice. **j,** Same as Fig. 3i for D2-MSNs. For the push action, 3g cross, late prep. and early prep. SVM peaked before peak force (see Extended Data Table 1, table ref. 3.33, 3.34, 3.35). The time of peak ensemble activity increased progressively across early preparation, late preparation, 3g cross, and force peak SVMs (see Extended Data Table 1, table ref. 3.36). Data are mean+s.e.m. from 78 sessions across 8 mice. **k,** Same as Fig. 3h for pull weighted neurons. For the pull action, 3g cross, late prep. and early prep. SVM peaked before peak force (see Extended Data Table 1, table ref. 3.37, 3.38, 3.39). The time of peak ensemble activity increased progressively across early preparation, late preparation, 3g cross, and force peak SVMs (see Extended Data Table 1, table ref. 3.40). Data are mean+s.e.m. from 86 sessions across 8 mice. **l,** Same as in Fig. 3i for pull weighted D1-MSNs. For the pull action, 3g cross, late prep. and early prep. SVM peaked before peak force (see Extended Data Table 1, table ref. 3.42, 3.43). The time of peak ensemble activity increased progressively across early preparation, late preparation, 3g cross, and force peak SVMs (see Extended Data Table 1, table ref. 3.44) . Data are mean+s.e.m. from 71 sessions across 8 mice. **m,** Same as in Fig. 3j for pull weighted D2-MSNs. For the pull action, 3g cross, late prep. and early prep. SVM peaked before peak force (see Extended Data Table 1, table ref. 3.45, 3.46, 3.47). The time of peak ensemble activity increased progressively across early preparation, late preparation, 3g cross, and force peak SVMs (see Extended Data Table 1, table ref. 3.48). Data are mean+s.e.m. from 84 sessions across 8 mice.

We observed that population activity predicted action identity well before action onset (force crossing 3g) (Fig. 3a, Extended Data Fig. 3). We compared the action identity decoder trained at force peak to decoders trained at each time lag. The dimensions of population activity that encoded action identity before execution were different from those at force peak (Fig. 3b), and decoders using these dimensions have higher accuracy (Fig. 3cd, Extended Data Fig. 3e). As time approached the force peak (e.g. 3g cross), the alignment increased between the instantaneous decoder and the decoder at force peak.

We identified an “early preparation” dimension from the SVM optimized at 140ms before 3g cross, a “late preparation” dimension from the SVM optimized on neural activity 70ms before 3g cross, a “3g cross” SVM dimension capturing action onset, and a “force peak” dimension capturing action execution. The projection of activity in those preparation dimensions peaked earlier than in the execution dimension for push (Fig. 3e) and pull actions (Fig. 3f). Further, the timing of peak activity increased progressively across the early preparation, late preparation, 3g cross, and force peak dimensions of population activity. Thus, striatal activity progressively modulates in preparation dimensions before execution (Fig. 3g).

Distinct ensembles of neurons participated in action preparation versus execution, consisting of both D1- and D2-MSNs. During the push action, push-weighted neurons in the preparatory dimensions peaked earlier than push-weighted neurons in the execution dimension (Fig. 3h), for both D1-MSNs (Fig. 3i) and D2-MSNs (Fig. 3j). Further, the timing of peak ensemble activity increased progressively across the ensembles identified from early preparation, late preparation, 3g cross, and force peak dimensions. We found the same effects for the pull-weighted neurons during the pull action (Fig. 3k–m). Thus, striatal D1- and D2-MSN ensembles progressively encode action preparation in population dimensions orthogonal to execution, emerging well before movement onset. Our finding shows that different striatal ensembles were modulated during preparation versus execution.

## Striatal ensembles encode action identity irrespective of reinforcement

**Fig 4:**
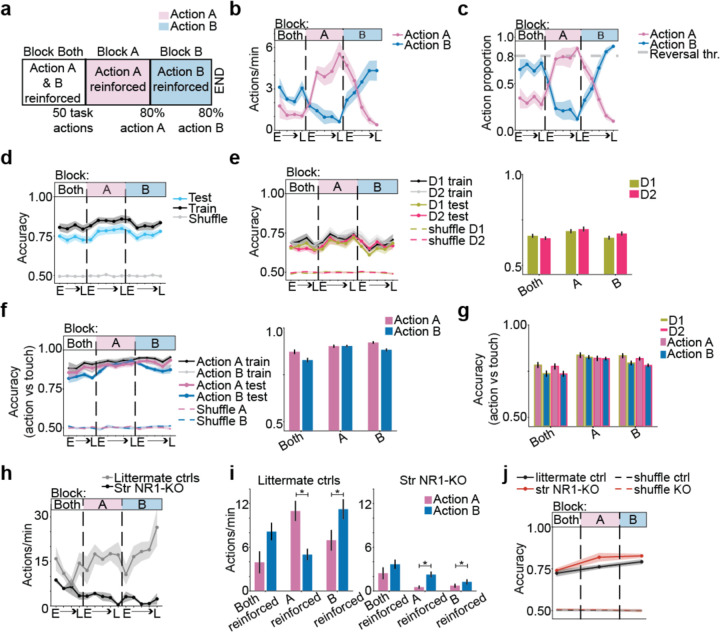
The striatum encodes isometric forelimb actions irrespective of reinforcement and plasticity. **a,** Schematic of reinforcement blocks. Criteria to advance to the next block are labelled. **b,** The rate of task actions (Methods). In Block Both, the rate of Action B was greater than Action A (see Extended Data Table 1, table ref. 4.1). In Block A, Action A rate increased with sessions (see Extended Data Table 1, table ref. 4.2), and Action B rate decreased with sessions (see Extended Data Table 1, table ref. 4.3). In Block B, Action B rate increased with sessions (see Extended Data Table 1, table ref. 4.5), and Action A rate decreased with sessions (see Extended Data Table 1, table ref. 4.4). **c,** The proportion of task actions from each identity (A or B). In Block A, the Action A proportion increased with sessions (see Extended Data Table 1, table ref. 4.7). In Block B, the Action B proportion increased with sessions (see Extended Data Table 1, table ref. 4.8). **d,** Decoding MSN activity at force peak to predict action identity. Accuracy was greater than shuffle on the first session (see Extended Data Table 1, table ref. 4.10) (paired t-test, statistic=7.40, *P*=1.5e-4), in pooled sessions from Block Both, Block A and Block B (see Extended Data Table 1, table ref. 4.11, 4.12, 4.13). **e,** Same as “d” but decoding D1- or D2-MSN activity. Right: average of sessions in each block. Accuracy was greater than shuffle in pooled sessions from Block Both, Block A and Block B (see Extended Data Table 1, table ref. D1: 4.16, 4.17, 4.18; D2: 4.19, 4.20, 4.21). There was no significant difference between D1- and D2-MSNs in each block (see Extended Data Table 1, table ref. 4.22–4.24). **f,** Decoding MSN activity at force peak to predict action A from touch and Action B from touch. Right: average of sessions in each block. Accuracy was greater than shuffle on the first session (see Extended Data Table 1, table ref. 4.25, 4.26) and in pooled sessions from Block Both, Block A and Block B (see Extended Data Table 1, table ref. A: 4.27, 4.28, 4.29; B: 4.30, 4.31, 4.32). **g,** Same as “f Right” but decoding D1- or D2-MSN activity. Decoding accuracy of action A versus touch and action B vs touch was greater than shuffle in pooled sessions from Block Both, Block A and Block B (see Extended Data Table 1, table ref. D1: 4.37–4.42; D2: 4.43–4.48). **b-g,** Data are mean+s.e.m. across n=8 mice for each session or for each block in bar plots. **h,** The rate of 6g cross actions (pooling push and pull) for control and mutant striatal NR1-KO mice. Data are mean+s.e.m. across n=4 control mice and n=3 NR1-KO mice for each session. For mutants, the action rate decreased with sessions (see Extended Data Table 1, table ref. 4.49). For control mice, the action rate increased with sessions (see Extended Data Table 1, table ref. 4.50). **i,** The rate of 6g cross actions in each block for control and mutant striatal NR1-KO mice. Littermate controls’ rate for each action was not significantly different when both actions were reinforced (see Extended Data Table 1, table ref. 4.51). Littermate controls performed action A more than action B when action A was reinforced (see Extended Data Table 1, table ref. 4.52) and action B more than action A when action B was reinforced (see Extended Data Table 1, table ref. 4.53). NR1-KO mice’ rate for each action was not significantly different when both actions were reinforced (see Extended Data Table 1, table ref. 4.53). NR1-KO mice performed action B more than action A when action A was reinforced and when B was reinforced (see Extended Data Table 1, table ref. 4.54, 4.55, respectively). **j,** Decoding MSN activity at force peak for littermate control and NR1-KO mice. Decoding accuracy was greater than shuffle in pooled sessions from Block Both, Block A and Block B (see Extended Data Table 1, table ref. NR1-KO: 4.56, 4.57, 4.58; controls: 4.59, 4.60, 4.61). **i-j,** Data are mean+s.e.m. over n=4 littermate control mice and n=3 NR1-KO mice, averaging sessions within each block.

The striatum has a classical role in learning to repeat specific actions that are reinforced, with D1- and D2-MSNs thought to have opponent^13,19^ or different^20^ roles. Thus, we next tested whether MSNs differentially encoded action identity in blocks where different actions were reinforced to determine if reinforcement altered the encodings in these two neuronal populations. We trained mice across three blocks of sessions, each with a different contingency for reinforcement (Fig. 4a, Extended Data Fig. 4ab, Methods). Even in the absence of external cues and overt movements, mice increased the frequency of the specific actions being reinforced. In the first block, both task actions, push and pull, were reinforced (Fig. 4bc - Block Both). Any given mouse defaulted to performing one of the actions more than the other, and we call that action the mouse’s *preferred action* (or Action B), and the other action the *least preferred* action (or Action A). Once mice performed enough actions during Block Both (Methods), Action A was reinforced during Block A. Mice increased the rate and proportion of Action A to Action B over Block A sessions (Fig. 4bc – Block A). Finally, once mice performed Action A much more than Action B (at least 80% of target actions for three consecutive sessions), we reversed the contingency, and switched Block B where only Action B was reinforced. Mice reversed their performance (Fig.4bc – Block B), increasing the rate and proportion of Action B until it was performed much more than Action A (see Methods).

We examined striatal action encoding across sessions, analyzing population activity at force peak for isometric actions that crossed 3g for greater than 50ms (3g cross actions, defined in Methods). Population activity predicted the identity of action well above chance across all reinforcement contingencies (Fig. 4d). Notably, population activity predicted action identity from the first session of training (Fig. 4d), and the encoding of each specific action did not decrease when only a single action was reinforced (Fig. 4d,f). Further, D1- and D2-MSNs encoded action identity with equal accuracy in each reinforcement block (Fig. 4e). Action identity was encoded across reinforcement even when matching trials for force, duration, and licking across actions (Extended Data Fig. 5, 6). These data indicate that striatal ensembles strongly encode the identity of ongoing actions independently of whether the particular action is reinforced or not.

Next, we asked whether the striatum encoded specific actions when striatal plasticity and reinforcement are impaired. To this effect, we trained and imaged striatal NR1-KO mutant mice (see Methods), which lack the essential NR1 subunit of N-Methyl-D-aspartic acid (NMDA) receptors, and have impaired long-term potentiation at glutamatergic synapses to MSNs^43^. Mutants were able to perform both isometric pushes and pulls (Extended Data Fig. 3e–g), but they did not increase the rate of action performance across reinforcement (Fig. 4g). They were unable to learn which action led to reinforcement (Fig. 4h, Extended Data Fig. 4g–i), as the rate of actions maintained its bias for Action B across reinforcement contingencies, even when only Action A was reinforced (Fig. 4h right). In contrast, littermate control mice increased overall action performance across reinforcement (Fig. 4g), and they learned the specific actions that led to reinforcement (Fig. 4h left, Extended Data Fig. 4cd, g–i), as observed previously. Nonetheless, action identity encoding in controls was not better than in NR1-KO mutants even though mutants showed no learning (Fig. 4j). Thus, striatal ensembles encoded action identity specifically and equally, not only across reinforcement contingencies, but also in the absence of plasticity, suggesting a role for D1- and D2-MSNs in executing specific actions irrespective of their role for reinforcement.

## Specific stimulation of striatal neurons encoding specific actions

**Fig 5:**
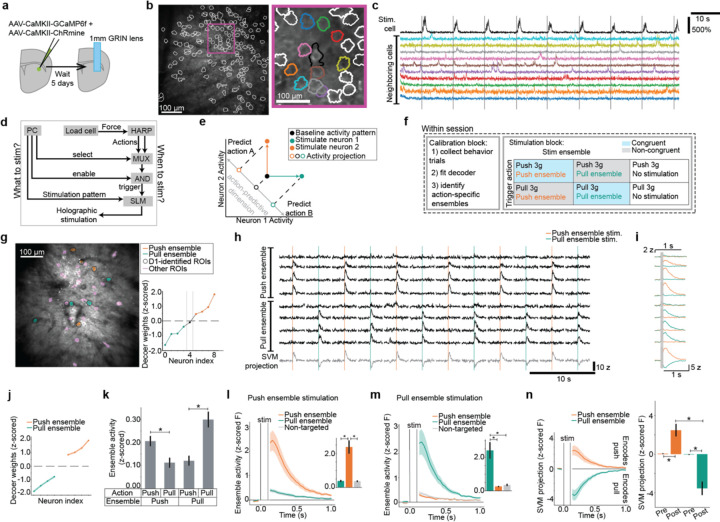
Closed-loop modeling and manipulation of neural ensembles that encode execution of specific actions. **a,** Schematic of GCaMP6f and ChRmine viral injection and lens implant in transgenic mice to image and photostimulate D1- and D2-MSNs. **b,** Example field of view with a single neuron targeted for stimulation. **c,** Example activity traces of a targeted neuron (in black) and neighboring non-targeted neurons during 2-photon optogenetics. **d,** Schematic of closed-loop system to select the pattern of holographic stimulation and the action that triggers stimulation. **e,** Cartoon depicting how activating specific neurons can manipulate a dimension of population activity that encodes and predicts specific actions. **f,** Schematic for conducting the modeling and manipulation experiment. **g,** Left: example field of view with regions of interest labeled based on their decoder weights and identification as D1-MSNs. Right: Decoder weights for identified D1-MSNs, colored by assignment into action-specific ensembles. **h,** Four consecutive stimulation trials from the beginning, middle, and end of the example session. **i,** Trial-averaged traces of targeted neurons and projection of population activity on the action-predictive SVM dimension. **j-n,** Data are mean+s.e.m. across n=21 sessions and 10 mice. **j,** Average decoder weights of action-specific ensembles. The top four neurons with the largest weights are shown. **k,** Average activity of action-specific ensembles in 100 ms before force peak during trials in the calibration block. Push ensemble activity was larger for push than pull (Extended Data Table 1, table ref. 5.1). Pull ensemble activity was larger for pull than push (Extended Data Table 1, table ref. 5.2). **l-m,** Neural activity averaged across neurons (z-scored fluorescence) within each ensemble, locked to stimulation of each ensemble. Inset: average ensemble activity in 100 ms after stimulation. **l,** After push ensemble stimulation, activity of the push ensemble was greater than the pull ensemble (Extended Data Table 1, table ref. 5.3) and non-targeted neurons (Extended Data Table 1, table ref. 5.4). There was no significant difference between the pull ensemble and non-targeted neurons (Extended Data Table 1, table ref. 5.5). **m,** After pull ensemble stimulation, activity of the pull ensemble was greater than the push ensemble (Extended Data Table 1, table ref. 5.6) and non-targeted neurons (Extended Data Table 1, table ref. 5.7). There was no significant difference between the push ensemble and non-targeted neurons (Extended Data Table 1, table ref. 5.8). **n,** Projection of population activity on the SVM dimension locked to ensemble stimulation. Inset: Average SVM projection in the 100 ms before stimulation (“pre”) and after stimulation (“post”). Stimulating the push ensemble increased the “post” SVM projection relative to “pre” (Extended Data Table 1, table ref. 5.9). Stimulating the pull ensemble decreased the “post” SVM projection relative to “pre” (Extended Data Table 1, table ref. 5.10). The “post” SVM projection was greater after push ensemble stimulation than pull ensemble stimulation (Extended Data Table 1, table ref. 5.11).

Given that D1- and D2-MSNs encode action preparation and execution irrespective of reinforcement, we then asked whether they control specific ongoing actions. One critical technical challenge was that we needed to target perturbations to specific and spatially-intermingled D1- and D2- MSNs that encode action identity specifically during execution. For this, we developed an approach to perform 2-photon optogenetics to target stimulation to individual MSNs through a GRIN lens^39^ in mice co-expressing GCaMP6f as well as the red-shifted opsin ChRmine^38^ (Fig. 5a–c). We used a spatial light modulator to holographically target a group of user-defined MSNs for simultaneous stimulation^37^. Another technical challenge was that the overall duration of action execution period was very short, and we needed to trigger stimulation upon self-paced and – initiated actions with flexibility and low latency, as force onsets rapidly over milliseconds (Fig. 1c, 3a). For this we developed an experimental platform that allowed us to flexibly select the trigger action and stimulation pattern and begin the stimulation with ~2ms total latency (Fig. 5d, Methods).

We used this system to manipulate population activity that encoded action identity during execution (Fig. 5e). Within a single session, we identified action-specific MSN ensembles and stimulated them in closed-loop during specific, self-paced actions (Fig. 5f). In a calibration block, we collected behavior trials and fit a decoder to predict action identity from MSN activity. As before (Fig. 2k–n), we identified action-specific ensembles (Fig. 5g) using the weights of the force peak SVM (the “execution dimension”, Fig 3g). We defined the action-specific ensembles as equally sized groups of 4–11 neurons with the largest decoder weights (mean ensemble size was 6.7 neurons over 21 sessions). Then in the stimulation block, specific self-paced actions triggered stimulation of action-specific ensembles (Fig. 5h,i). We interleaved 6 conditions: two actions that triggered stimulation (3g push, 3g pull) and three stimulation patterns for each action (push ensemble, pull ensemble, no stimulation) (Fig. 5f).

Our experiments identified action-specific ensembles with similar magnitude decoder weights (Fig. 5j) and that modulated specifically for their corresponding actions (Fig. 5k). Stimulation specifically activated the targeted ensemble (Fig. 5l,m). We verified that this stimulation of a small number of action-specific neurons bi-directionally drove the action-specific SVM dimension of population activity (Fig. 5n). Thus, selective stimulation of small, action-specific striatal ensembles was sufficient to bias population activity toward encoding the corresponding action.

## Specific striatal ensembles control specific actions

**Fig 6:**
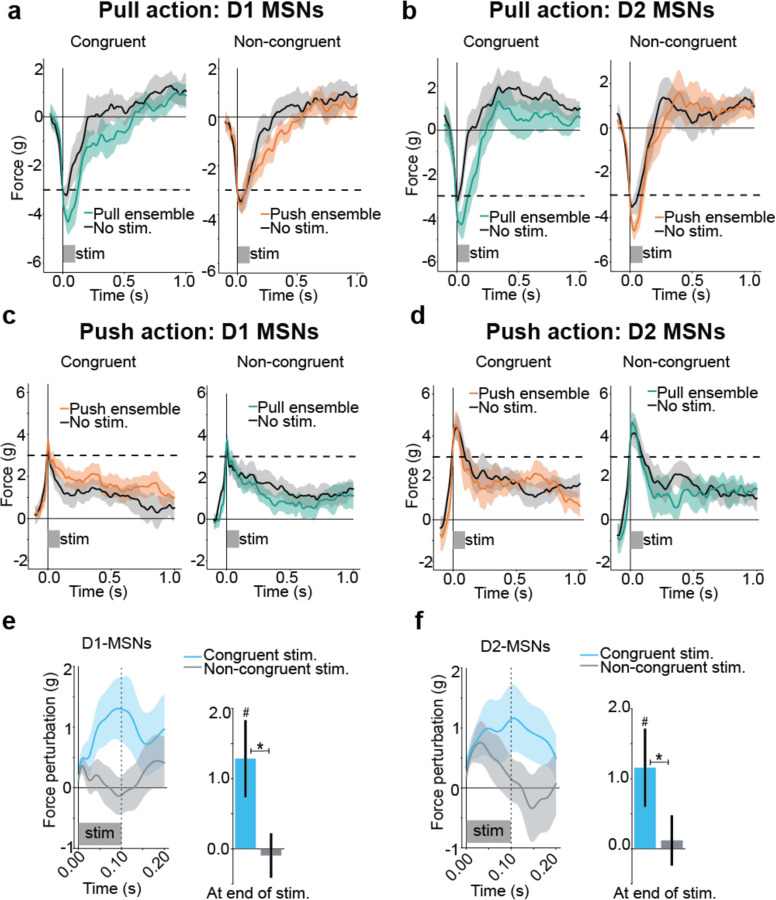
Action-specific ensembles of D1- and D2- MSNs control congruent actions. **a,** Trial-averaged force traces locked to pull action that triggers photostimulation of a D1-MSN ensemble. Data are mean+s.e.m. across n=12 sessions and 9 mice. **b,** Same as “a” except triggering D2-MSN ensemble stimulation. Data are mean+s.e.m. across n=9 sessions and 5 mice. **c,** Trial-averaged force traces locked to push action that triggers photostimulation of a D1-MSN ensemble. Data are mean+s.e.m. across n=12 sessions and 9 mice, same as “a”. **d,** Same as “c” except triggering D2-MSN ensemble stimulation. Data are mean+s.e.m. across n=9 sessions and 5 mice, same as “b”. **e-f,** Force perturbation, i.e. the trial-averaged force trace on stimulation trials minus non-stimulation trials. By convention, positive perturbation increases the force of action. Right panel is the average perturbation during at the end of 100 ms of stimulation. **e,** Force perturbation from stimulation of D1-MSNs. Congruent force increased with congruent stimulation (Extended Data Table 1, table ref. 6.15) and was significantly different with non-congruent stimulation (Extended Data Table 1, table ref. 6.14) at the end of stimulation. Non-congruent was not significantly different than no stimulation (Extended Data Table 1, table ref. 6.16). **f,** Force perturbation from stimulation of D2-MSNs. Congruent force increased with congruent stimulation (Extended Data Table 1, table ref. 6.31) and was significantly different with non-congruent stimulation (Extended Data Table 1, table ref. 6.30) at the end of stimulation. Non-congruent was not significantly different than no stimulation (Extended Data Table 1, table ref. 6.32).

We used this manipulation approach to test whether striatal activity influences ongoing actions, and whether action-specific striatal ensembles control the execution of specific actions or just invigorate any ongoing action. This also allowed us to test whether D1-MSNs promote and D2-MSNs suppress movement, or both promote movement. During ongoing pull actions, stimulating the D1-MSNs pull ensemble increased the pull force (Fig. 6a left) while stimulating the push ensemble did not have the same effect (Fig. 6a right). Notably, for D2-MSNs, stimulating the pull ensemble during pull actions also appeared to increase pull force (Fig. 6b). Converse effects were observed when stimulating during the push action (Fig. 6c, d). We refer to the pairing of an action with ensemble stimulation specific to that action as “congruent” (i.e. push ensemble stimulation during push action, and pull ensemble stimulation during pull action), and the pairing of an action with ensemble stimulation specific to the other action as “non-congruent” (i.e. pull ensemble stimulation during push action, and push ensemble stimulation during the pull action) (Fig. 6). We quantified the force perturbation as the difference between force traces in stimulation trials and non-stimulation trials, plotting congruent force as positive and non-congruent force as negative. We found that at the end of the 100ms stimulation window, stimulation of an action-specific ensemble increased congruent force but not non-congruent force for both D1- and D2-MSNs (Fig. 6e,f).

## Discussion

In this study, we show that striatal ensembles can specifically encode—and causally control—minimally different actions of the same effector. Further, striatal population activity encodes ongoing forelimb actions even when those actions are not reinforced, and further, when reinforcement and learning is disrupted through impaired plasticity to the striatum. Although the striatum is often viewed as being permissive, triggering actions prepared elsewhere, we find that it also encodes action preparation well in advance of movement onset, in a subspace that is distinct from execution. Both D1- and D2-MSNs encode preparation and execution with equal accuracy, challenging the classical view that they promote versus suppress movement. We investigated if these ensembles were causally involved in the execution of ongoing actions by developing a closed-loop system that allowed us to identify and manipulate small groups of striatal neurons that encode execution of specific actions. These manipulation experiments revealed that both D1- and D2 action-specific ensembles control the execution of the corresponding ongoing actions. Despite the basal ganglia’s indirect access to spinal circuits, these results reveal that the striatum exerts fine-grained, online control over specific actions and forces, highlighting a previously unappreciated role of the striatum as a motor center in the brain.

Interestingly, striatal ensembles encode action identity through separable dynamics for action preparation and execution, and are not simply labelled lines for action with ramping activity. Population activity encoded the identity of upcoming action well before execution with different activity dimensions and neural ensembles than execution (Fig. 3), reminiscent of output-null preparatory activity in motor cortices^29,33,44,45^. Unlike the cue-driven paradigms typically used to study cortical preparation^46^, our self-paced task reveals long-timescale (>0.5 s) preparatory dynamics. These findings challenge models in which the basal ganglia merely provide “on/off’’ triggers^27,41,42^, and instead support a role for the basal ganglia in distributed population dynamics that prepare and execute volitional movement^30^.

These experiments dissociate the striatum’s role in encoding and controlling ongoing actions from its role in reinforcement learning. Dopaminergic input modulates plasticity at cortico-striatal synapses^18,47^, encodes movement outcomes^48,49^, and reinforces movements^18,50,51^ and neural patterns^52^ so that they increase in frequency. Although bulk striatal stimulation can reinforce preceding actions^13,19,20^, we find that striatal ensembles encode fine-grained forelimb actions independent of which action is reinforced (Fig. 4d,f). Strikingly, this encoding persists even when plasticity at glutamatergic synapses to the striatum is impaired^43^. These results suggest that the striatum has a function for motor control that is dissociable from its function in learning, and that learning from reinforcement makes use of striatal encoding of action space.

These results also offer new views on the canonical model that D1- and D2-MSNs respectively promote and suppress movement^3,9,25,28^ via the striatonigral and striatopallidal pathways. While bulk manipulations have supported opposing effects^13,25^, subsequent manipulations^35,36^ suggested that their coordinated activity are necessary for movement execution. Here we identified and stimulated action-specific ensembles of D1- and D2-MSNs and found that both increase the force of congruent actions. Thus, D2-MSNs can not only promote movement, but also control specific actions in real time, online. This finding suggests that D2-MSN projections to GPe can have effects that are independent of canonical inhibition of GPi/SNr, possibly through different anatomical circuits linking GPe directly to thalamus, cortex and brainstem^53^. It is also important to note that we focused our recordings and manipulations on forelimb region of dorsolateral striatum, and understanding the function of D1- and D2-MSNs would likely depend on the striatal subregion^28^, each receiving distinct topographic inputs from cortex^54,55^ and thalamus, and also projecting to specific outputs. Finally, the faster onset of perturbations evoked by D1-MSN congruent stimulation suggests different roles for D1- and D2-MSNs in the control of movement.

This study positions the striatum as a center for controlling granular ongoing actions, despite its indirect connections to the spinal cord. Although basal ganglia outputs tonically suppress downstream motor centers^27,56–58^, recent work reveals highly specific output dynamics during forelimb behavior^59^. Our work provides a foundation for future work on a fundamental problem: to understand how action-specific ensembles in the center of the brain can have such remarkably specific control over downstream motor centers. Conceptually, we hypothesize that the striatum guides motor centers into specific subspaces of neural population dynamics which implement action plans and execution^60^, consistent with evidence that striatal activity^61^ and corticostriatal plasticity^62^ are required for the brain to learn and control low-dimensional cortical dynamics^52,63,64^ that directly operate brain-machine interfaces.

The discovery that striatal ensembles control granular actions offers a framework for understanding movement disorders with striatal dysfunction characterized by impaired, highly specific motor patterns—such as task-specific dystonias^65^ (e.g. writer’s cramp, musician’s dystonia) and the choreic disruptions of selective movements observed in Huntington’s disease^2,9^.

## Methods

### Animals

All experiments and procedures were performed according to National Institutes of Health (NIH) guidelines and approved by the Institutional Animal Care and Use Committee (IACCUC) of Columbia University. Adult male mice, aged 2–6 months, were used for these experiments. For imaging and holographic stimulation experiments we labeled D1-MSNs and D2-MSNs with the following transgenic lines (more details in Extended Data Table 1): Tg(Drd1-cre)EY217Gsat, Tg(Drd1-cre)EY217Gsat crossed with Ai9(RCL-tdT), Tg(Adora2a-cre)KG139Gsat, Tg(Adora2a-cre)KG139Gsat crossed with Ai9(RCL-tdT) and Drd1a-tdTomato line 6. For experiments testing plasticity we used the following lines (more details in Extended Data Table 1): RGS9-cre crossed with NMDAR1-loxP ^43^. For comparison, we used the negative Cre littermates as controls. Mice used for experiments were individually housed and kept under a 12-h light-dark cycle.

### Randomization and blinding

The experimenter was blinded to the experimental groups for plasticity experiments. All animals were run on each day in a fixed random order. Holographic manipulations were performed according to a pre-defined protocol and controlled by Matlab in closed-loop with the mouse’s behavior - not manually by the experimenter. Sessions for stimulating D1- and D2-MSNs were interleaved as much as possible.

### Surgeries

For imaging of neurons in dorsolateral striatum (DLS), the viral injection and lens implant occurred in 2-step surgery: viral injection at least 5 days prior to lens implant. Viral injection and lens implanted follow protocols as described in protocols.io (“Viral injection” and “Lens implant:). For imaging, a total of 300 nl of AAV5.CaMKII.GCaMP6f (titer: 1e12–1e13 GC/ml) was injected into the left DLS of 2–6 month old mice. For holographic optogenetics experiments, an additional 300 nl of AAV8.CaMKII.ChRmine (titer on the order of 1e11 GC/ml) was injected with AAV5.CaMKII.GCaMP6f (ratio 1:1) into the left DLS. Coordinates for viral injections were: AP: +0.75 mm, ML: 2.5 mm, DV: 2.4 mm. Coordinates for lens implant were same as viral injections except DV: 2.0mm. To measure forelimb muscles’ activity, we implanted electromyographic electrodes (EMGs) after lens implant and attached the EMG connector to the caudal edge of existing implant. These EMGs were produced in house^66^ and implanted as described in protocols.io (“EMG_implants”).

### Histology

Histology was performed as described in protocols.io (Histology) to verify lens coordinates and viral expression. To localize imaging site, immunohistochemestry for GCaMP-GFP expression was performed by incubating the sections with Alexa 488nm conjugate GFP antibody at 1:1000 in 0.4% Triton-PBS overnight at room temperature. To identify tdTomato-somatic expression, immunohistochemistry for tdTomato was performed by incubating the sections with a primary anti-RFP antibody at 1:2000 0.4% Triton-PBS overnight at 4°C followed by secondary antibody anti-Rabbit Alexa Fluor 647 at 1:1000 0.4% Triton-PBS at room temperature for 2 hours. All slices were counterstained with DAPI at 1:1000 – excitation at 405 nm. Sections were imaged using an AZ100 automated slide scanning microscope equipped with a ×4 0.4-NA objective (Nikon) within the Zuckerman Institute’s Cellular Imaging Platform (RRID:SCR_027061).

### Two-action isometric task

#### Setup

Mice were head-fixed and positioned in a 3D-printed cup^67^ with a sucrose spout within tongue reach. The right forelimb interacted with an immobile joystick (3 mm screw) mounted on 3D-printed frames attached to two load cells (Phidgets 3139_0) measuring push–pull and left–right forces. The left forelimb rested on an auxiliary pole equipped with two additional load cells measuring push–pull and vertical forces. The body-support cup was mounted on a fifth load cell to capture overall body movement. Signals from all load cells were sampled at 1 kHz using the Champalimaud Foundation Scientific (CFS) “Scientific Board v1.3” with “Quad Load Cell v1.2” module, which also detected threshold crossings. Data were visualized and stored using the CFS “Load Cells Visualizer” software.

A pyControl “Lickometer v1.0” detected licking and joystick/pole contact. Video was acquired at 30–60 fps (Teledyne FLIR Flea3 1.3 MP, Mono: FL3-U3–13Y3M-C) and controlled with Bonsai software^68^. Task control and sucrose delivery were managed by a pyControl^69^ “Breakout 1.2” board, which logged events at 1 kHz and opened a solenoid to dispense ~5 µl of 10% sucrose solution via gravity-fed tubing and a blunt 16G spout. The solenoid timing was calibrated daily, and sucrose solution was prepared weekly. Hardware schematics are shown in Extended Data Fig. 7a.

#### Training

Mice were habituated to head fixation for 15 min on a running wheel over four days and then food-restricted to 80–85% of initial body weight. Training for the two-action isometric task was as follows (see protocols.io for detail, “Behavior training”). In this self-paced task, mice pushed or pulled an immobile, pressure-sensitive joystick. A task action was defined as a force exceeding a task-defined threshold for longer than the task-defined duration. Thresholds, durations, and reinforcement delays were progressively adjusted during training (Extended Data Table 2). Reinforcement followed the end of a valid action after a delay, while a 3 g quiescence threshold defined trial initiation.

Each trial consisted of: (1) a 1 s inter-trial interval (ITI); if forces were below 3 g at 1 s, a new trial began, otherwise the ITI reset; (2) a trial period, during which a task action (push or pull exceeding the threshold for the task duration) triggered delayed sucrose delivery. Trials terminated upon reinforcement or when force fell below threshold after an invalid (too brief) action.

Training comprised three sequential blocks with distinct reinforcement contingencies. In Block Both, both push and pull actions were reinforced. On the last session, the less frequent action was defined as action A and the more frequent as action B. In Block A, only action A was reinforced; in Block B, only action B was reinforced. Block Both sessions ended after 50 push and 50 pull task actions or 30 min. Blocks A and B ended after 200 reinforced actions or 30 min. Task parameters advanced to the next level (Parameters 1→2→3; Extended Data Table 2) when mice executed >50 valid task actions in a session. Block Both concluded when mice exceeded 50 actions in two consecutive sessions at Parameters 3. Blocks A and B each concluded when the reinforced action reached ≥80% of total actions over three consecutive sessions at Parameters 3.

For Rgs9-LCre::Grin1tm1Yql homozygous mice (Striatal NR1-KO) and Cre-negative littermates, the same training protocol was used. The maximum number of sessions per block was capped at 1.2 × the duration required by the third fastest mouse to reach the reinforcement-change criterion. “Give-up” criteria were defined as <10 3 g threshold crossings (≥50 ms duration) averaged over three consecutive sessions. Animals meeting either criterion advanced to the next training phase.

#### Selected sessions for analysis of behavioral training

Because animals were trained to criterion, the number of sessions per block varied across individuals. To enable cross-animal comparisons, specific sessions were selected for analysis. In Block Both, four sessions were chosen, and in Blocks A and B, five sessions were chosen, following the selection rules detailed in Extended Data Table 3.

#### 2-photon microscope

This is described in detail in Supplementary Information. The optical setup included two femtosecond lasers and a custom-modified two-photon microscope (Ultima In Vivo, Bruker) with an 8 kHz resonant scanner. Imaging used a Ti:sapphire laser (Chameleon Vision-S, Coherent) tuned to 920 nm for GCaMP6f, with power controlled by a Pockels cell (Conoptics). Photostimulation used a 1 MHz amplified laser (Monaco 1035–40, Coherent) at 1035 nm, controlled by an integrated acousto-optic modulator and directed through a spatial light modulator (Bruker Neuralight 3D) for holographic stimulation.

Imaging and stimulation were controlled by PrairieView (Bruker) and custom MATLAB/Python software interfacing via the PrairieLink API. Custom software defined stimulation patterns and triggered closed-loop photostimulation based on behavioral events.

The typical imaging power was < 50 mW, and could be up to 80 mW for deeper than ~ 250 μm below the terminus of the GRIN lens. Images were acquired using Prairie View software (Bruker Corporation, Billerica, Massachusetts) at 30 Hz. The functional data was extracted from a square of 512 x 512 pixels of the following size: for Fig. 1–4 we used 1.5x zoom over 496.1 μm x 496.1 μm; in Fig. 5–6 we used 1.5x zoom over 550.4 μm x 550.4 μm and 2x zoom over 412.7 μm x 412.7 μm.

#### EMG recordings

EMG recordings were performed as described in protocols.io (“EMG recordings”). Custom-made electrodes were implanted in forelimb muscles and connected to an Omnetics connector. Signals were acquired via a 16-channel TDT ZC16 headstage connected to a TDT RZ5D Bioamplifier and PZ5–32 preamplifier. Muscle activity was recorded in true differential mode at 24 kHz. For analysis, EMG signals were downsampled to 1 kHz, high-pass filtered at 40 Hz, rectified, and smoothed with a Gaussian kernel (SD = 25 ms) ^70^. Data included 41 recording sessions from 4 mice performing the two-action isometric task (Fig. 1), from a cohort distinct from the 8 mice shown in Figs. 2–4.

### Adaptive Isometric task

#### Setup

Described in detail in Supplementary Information. The setup was identical to the two-action isometric task, except that hardware was added to flexibly select force-threshold crossings to trigger holographic stimulation (Extended Data Fig. 7b). Force thresholds were monitored in parallel using an updated load-cell system (“HARP load cells acquisition v1.1” and “HARP load cell interface v1.1,” CFS Hardware Platform). A custom circuit containing a MUX and AND gate routed trigger signals to the microscope. Custom MATLAB scripts controlled the MUX and AND gate via a NIDAQ USB-6001 board, specifying which threshold triggered stimulation and when triggers were enabled.

#### Training

This task was used for holographic stimulation experiments and followed the two-action forelimb task with modified reinforcement to promote balanced performance of both actions. Mice were trained for 3 weeks with adaptive reinforcement and no stimulation, partly in behavior boxes without imaging. Reinforcement probability was adjusted online based on performance: the proportion of push and pull actions was tracked over the last 10 trials. When both actions occurred in <70% of recent trials, both were reinforced; if one exceeded 70%, that action was unrewarded.

#### Protocol to image the same field of view and neurons across sessions

For holographic stimulation experiments, the same field of view and neurons were imaged across paired sessions. After the first session, imaging data were processed with Suite2p^71^ to extract active ROIs. In the paired session, PrairieView’s “Brightness over Time” (BOT) feature was used to annotate these neurons in the GUI. Imaging power was kept constant by continuously measuring the laser after the Pockels cell and adjusting its bias. Detailed methods are in protocols.io (“2-photon imaging”).

#### Calibration of 2-photon stimulation spatial targeting and power

Spatial targeting of 2-photon stimulation was calibrated before each session (protocols.io, “2-photon stimulation calibration”). Using PrairieView’s “burn spots” routine, we iteratively aligned burned spots on a fluorescent sample to target locations, allowing the software to calibrate galvos’ control for accurate stimulation. Calibration was stable across sessions, requiring only minor adjustments.

Before experiments, we calibrated stimulation power and duration for each ROI. Using custom Python scripts with PrairieView’s “Brightness over Time” (BOT) feature, we visualized ROI responses while sweeping power (3–7 mW per target) and duration (50–100 ms) to identify the minimum parameters that reliably activated the targeted neurons.

#### Analysis

Analysis was performed using custom scripts in Python.

#### Data extraction and synchronization

All data streams—task events (1 kHz), joystick forces (1 kHz), EMG (24 kHz), 2-photon imaging/voltage (30 Hz/1 kHz), and video (30–60 Hz)—were synchronized via the ITI signal, providing a unified timestamp across modalities for precise alignment of behavior, forces, EMG, imaging, and video.

#### Calibrated force measurement

The joystick voltage signal was converted to force using a linear model: Force (g) = (Voltage – baseline) × conversion factor. Baseline voltage was measured before each session and defined as the average voltage with no weight on the sensors. The conversion factor was determined by applying known weights (0, 2, 5, 20 g) and fitting a linear model. Conversion was stable and re-calibrated weekly to correct drift.

#### Definition of actions

For analysis, we defined several actions from force and touch signals. Task actions were push and pull events identified by the hardware from 1 kHz force traces, with intervals from threshold rise to fall. 3 g cross actions were pushes or pulls exceeding 3 g for ≥66 ms with joystick touch ≥100 ms before crossing to ensure isometric behavior; force traces were downsampled to 30 Hz. 6 g cross actions were a subset of 3 g crosses peaking above 6 g, the final training threshold. Touch was defined as joystick contact <2 g for >1 s, and licks as ≥5 licks with <0.5 s inter-lick interval, excluding overlap with 3 g crosses. We also analyzed special subsets: pre-isolated 3 g crosses had no other 3 g crosses in the 0.5 s before, post-isolated 3 g crosses had none in the 0.5 s after, and matched actions were push/pull trials matched for features (see “Matching trials across actions”). See Supplementary Information for more details.

#### Action events

We analyzed the following action events. For push and pull actions, analysis was aligned to two key points within the action interval: the moment the force threshold was crossed and the moment of peak force. For touch and lick actions, analysis was aligned to the midpoint of the action interval.

#### Matching trials across actions

We tested whether neural activity encoded push and pull actions with matched trial-averaged features (Fig. 1f–j, Extended Data Fig. 5,6). Tolerances were: average force ≤0.1 g (Fig. 1f–j); peak force ≤0.1 g, two-axis force magnitude at peak ≤0.2 g, action duration ≤30 ms, and lick rate/probability during actions and at peak ≤0.5 Hz and ≤0.1, respectively (Extended Data Fig. 5,6).

Trials were iteratively removed to achieve matching while minimizing loss of trials. Features were z-scored, then for each iteration: (1) define action 1 as the one with fewer trials, action 2 as the other; (2) compute the target vector as the difference in mean features; (3) zero in-tolerance features; (4) compute dot products of action 2 trials with the target; (5) remove the trial with the smallest dot product; (6) repeat until all features met tolerance.

For Extended Data Fig. 5,6, trials were pre-selected so solenoid opening preceded force threshold crossing by ≥4 s, excluding sucrose-related actions. See Supplementary Information for more details.

#### Time course of action force

Trial-averaged joystick force was computed as peri-event time histograms (PETHs) locked to action events: 6 g cross push/pull actions were aligned to 6 g cross (Fig. 1c), 3 g cross actions to the force peak (Fig. 2c) or 3 g cross (Fig. 3a, Extended Data Fig. 3). In Fig. 2f/Extended Data Fig. 3c, −4–0 s shows pre-isolated and 0–4 s shows post-isolated 3 g cross force, illustrating trials used to predict action identity from neural activity. See Supplementary Information for more details.

#### Quantification of actions across reinforcement

Action performance was quantified across the reinforcement schedule. For 6 g cross actions, we quantified action rate pooled over each reinforcement block, and overall rate for each session (Fig. 1d, 4h,i, Extended Data 4g). For task actions, we quantified action rate and action identity proportions across sessions (Fig. 4b,c, Extended Data 4a–f,h,i). For 3 g cross actions, we quantified number of actions, peak and average force, two-dimensional force magnitude, average lick probability, action duration, action rate, and action proportion (Extended Data Fig. 5a–h, k–r). See Supplementary Information for more details.

#### EMG analysis

We analyzed 1 kHz filtered EMG from four forelimb muscles for 6 g cross actions with force matched within 0.1 g. EMG channels were z-scored across each session and aligned to the 6 g cross event. To capture dominant muscle activity patterns, we performed PCA (sklearn.decomposition.PCA) on a 10 s window of trial-averaged force per action, forming a 4 muscles × (2 actions × 10,000 samples) matrix, and analyzed projections onto the first principal component (PC1, Fig. 1h). EMG similarity was quantified using a 0.4 s window: the mean across actions defined “average modulation,” and half the difference defined “differential modulation” (Fig. 1i). Action specificity was assessed by comparing EMG vector differences across actions versus within-action trial halves averaged over 100 ms around 6 g cross (Fig. 1j). See Supplementary Information for more details.

#### Neuron detection and activity extraction with Suite2p

2-photon imaging data were processed with Suite2p^71^ for motion correction, neuron detection, and fluorescence extraction; ROIs were classified using a classifier trained on sparse striatal MSN activity, neuropil-corrected (F_corrected_ = F – 0.7 x F_neu_ ), and neural activity reported as z-scored ΔF/F_0_ with F_0_ as the 1-minute running median, while Fig. 5 shows raw fluorescence from PrairieView’s “Brightness over time” (BOT).

#### Distinguishing D1- and D2-MSNs

We developed an automated thresholding method to classify D1- and D2-MSNs using red-channel fluorophore co-localization with functional ROIs identified via Suite2p (green channel), validated against manual labeling (ground truth); structural and functional images were aligned and processed (Extended Data Fig. 1a), and analyses and holographic stimulation experiments used the automatic method, which closely matched manual labels. See Supplementary Information for more details.

#### Time course of neuronal activity

Neuronal activity was analyzed relative to action events. Activity averaged over trials and all MSNs or a random subset of D1- and D2-MSNs is shown (Fig. 2b). PETHs aligned to the force peak of pre-isolated 3 g cross actions are shown for all MSNs (Fig. 2c) and example individual D1- and D2-MSNs (Fig. 2d). See Supplementary Information for more details.

#### Support Vector Machine (SVM) decoder model

To test whether MSNs encoded specific actions, we trained a linear SVM (sklearn.svm.LinearSVC) to predict action identity from single-trial population activity. Each trial vector comprised the ΔF/F₀ of each neuron averaged over 5 frames (~167 ms) centered on the action event (i.e. we used the frame locked to a time point and 2 frames before and after) (Fig. 2h,i; Extended Data Figs. 5,6). Only sessions with ≥20 trials per action and ≥10 neurons were analyzed, and trials were weighted by 1/number of trials to prevent bias. Trials were randomly split 90/10 for training/testing, repeated 100 times. The regularization parameter (C) was optimized via 10 sub-folds of the training set. Accuracy was computed overall and for each action separately, and compared to a shuffle control trained on randomized labels. See Supplementary Information for more details.

#### Matching number of D1- and D2-MSNs for SVM decoding

To compare D1- and D2-MSNs, we equalized their numbers for SVM decoding (Extended Data Fig. 6a,b). For the cell type with more neurons, a random subset was selected in each training/testing fold to match the number of neurons in the other cell type.

#### SVM decoding across sessions

We reported the accuracy of decoding action identity for individual sessions across reinforcement and pooling sessions. See Supplementary Information for more details.

#### Time course of action decoding

We used the SVM to analyze the time course of action decoding. We extracted and decoded neural activity centered at specific time points relative to action events. See Supplementary Information for more details.

#### Overlay of decoder accuracy and force

Decoder accuracy and force were visualized in normalized units by centering data at a reference time point and scaling by the window’s maximum trial-averaged value. See Supplementary Information for more details.

#### SVM weights and dimension

The SVM assigned positive (negative) weights to neurons whose activation biased the SVM to predict push (pull). For analysis of the weights directly, we z-scored the weights for each decoder (Fig. 5g,j, Extended Data Fig. 2f). The weights define a dimension of population activity that best discriminates action identity. We extract this dimension using the decoder’s weight vector and dividing by its L2 norm. This is a vector $w\in R^{N}$, where N is the number of neurons and the vector magnitude is 1 $\left({\left\|w\right\|}_{2}=1\right)$.

#### Time course of SVM projection

We visualized action-specific population activity by projecting the activity vector $x\in R^{N}$ (where each entry is one neuron’s activity) onto the SVM dimension w. At each time point, the projection $w^{T}x$ gives a scalar representing the weighted sum of neuronal activity. See Supplementary Information for more details.

#### SVM-weighted neural ensembles

We defined neuron ensembles that biased the SVM toward push or pull. For each action, we selected the top k * ensemble_frac neurons with the largest positive (push) or negative (pull) weights, where k is the smaller of the number of push- or pull-weighted neurons. We used ensemble_frac = 0.25 to visualize the most strongly weighted neurons (Figs. 2k–n, 3h–m) and ensemble_frac = 1 to select as many neurons as possible for holographic optogenetic stimulation (Figs. 5g–n).

#### Time course of SVM-weighted neural ensembles

We analyzed the activity of push weighted and pull weighted neural ensembles that biased the SVM to predict push or pull. See Supplementary Information for more details.

#### Decoder alignment

We quantified decoder alignment using the subspace angle between SVM weight vectors (“scipy.linalg.subspace_angles” in Python). An angle of 0° means the decoders use the same population dimension, and 90° means orthogonal dimensions. In Fig. 3b, we compared the force-peak SVM to early preparation, late preparation, and 3g cross SVMs.

#### Activation timing

We measured activation timing as when SVM projections or weighted ensembles crossed a percentile threshold relative to force peak. In Fig. 3ef, we reported the time when the SVM projections crossed the 99^th^ percentile in a 2 second window centered at force peak of pre-isolated 3g cross actions. (For the pull action, we negated the projection for this calculation, since the SVM projection decreases leading to force peak.) In Fig. 3h–m, we reported the time when push weighted and pull weighted ensemble activity crossed the 90^th^ percentile for the same time window and actions.

#### Holographic optogenetics experiments

We used holographic optogenetics to test how stimulating specific neuronal ensembles affects self-paced forelimb actions. Experiments relied on paired sessions imaging the same field of view. In the first session, we performed 2-photon imaging during the adaptive isometric task, extracted ROIs with Suite2p, and labeled D1- and D2-MSNs. In the second session, we ran stimulation experiments with a calibration block and a stimulation block (Fig. 5f). Stimulation targeted D1- or D2-MSNs based on session balance and available neurons. In the calibration block, we z-scored neuronal fluorescence and used an SVM to predict push vs. pull. Neurons with the largest SVM weights formed push- and pull-weighted ensembles, whose activity was verified before stimulation. In the stimulation block, holographic stimulation targeted ensemble ROIs (3–7 mW per neuron, 100 ms, 5 spiral traces) triggered by force threshold crossings. Trials were pre-scheduled and interleaved: stimulation was triggered by either 3g cross push or pull, and patterns included push ensemble, pull ensemble, or no stimulation. Each block had six trials in the order: no stim, push, no stim, no stim, pull, no stim, with 2 s inter-trial intervals. Our custom setup (Fig. 5d, Extended Data Fig. 7b) enabled ~2 ms latency stimulation triggered by specific force crossings. See Supplementary Information for more details.

#### Effect of holographic stimulation on actions

We compared force in stimulation trials to the preceding no-stimulation trials, excluding those closer to the previous stimulation than the next. Force was sampled at 1 kHz and low-pass filtered at 30 Hz (10th-order Butterworth).

To account for variable stimulation latency, no-stimulation trials were aligned to stimulation by matching instantaneous force at stimulation and average force in the preceding 100 ms.

We analyzed stimulation versus aligned no-stimulation trials pooled into “congruent” (push→push, pull→pull) and “non-congruent” (push→pull, pull→push) conditions. Data points for statistics were 2× num_sessions. See Supplementary Information for more details.

#### Statistical analysis

Statistical analysis was performed using custom scripts in Python. We used the ‘scipy.stats’ package to perform paired t-tests (‘ttest_rel’ method), unpaired t-tests (‘ttest_ind’ method), and linear regression (‘linregress’ method). Linear mixed effects models were performed using the ‘statsmodels’ package and ‘mixedlm’ method. Significance was set at P=0.05. See Supplementary Information for more details.

## Supplementary Material

Supplement 1

Supplement 2

Supplement 3
