# Supplementary material for: Striatal ensembles specify and control granular forelimb actions
Source: bioRxiv. 2025 Dec 8:2025.12.03.692128. Preprint. [Version 1] doi: 10.64898/2025.12.03.692128 (PMC12713657; doi:10.64898/2025.12.03.692128)
Supplement: Supplement 1 [file media-1.docx]

**Extended Data**

| Line name | Extended name | Source | Available at | Code | Ref. |
| --- | --- | --- | --- | --- | --- |
| Tg(Drd1-cre)EY217Gsat | STOCK Tg(Drd1-cre)EY217Gsat/Mmucd | Gerfen lab* | MMRRC | 030778 | Gong et al, 2007 |
| Ai9(RCL-tdT) | B6;129S6-Gt(ROSA)26Sortm9(CAG-tdTomato)Hze/J | Jackson Laboratories | Jackson Laboratories | 007905 | Madisen et al, 2010 |
| Drd1a-tdTomato line 6 | B6.Cg-Tg(Drd1a-tdTomato)6Calak/J | Jackson Laboratories | Jackson Laboratories | 016204 | Ade et al, 2011 |
| Tg(Adora2a-cre)KG139Gsat | B6.FVB(Cg)-Tg(Adora2a-cre)KG139Gsat/Mmucd | MMRRC* | MMRRC | 036158 | Gong et al, 2007 |
| RGS9-cre | B6;129S-Rgs9^tm1.1(cre)Yql^/J | NIH from Yuqing Li* | Jackson Laboratories | 020550 | Dang et al, 2006 |
| NMDAR1-loxP | B6.129S2(Cg)-*Grin1^tm1Yql^*/NkzaJ | NIH from Yuqing Li* | Jackson Laboratories | 036352 | Dang et al, 2006 |

* These lines were kept in house and backcrossed for more than 10 generations.

Table 1 – Mouse lines used in experiments.

| Reinforcement block | Parameters label | Task action threshold (g) | Task action duration (ms) | Reinforcement delay (ms) |
| --- | --- | --- | --- | --- |
| Both | 1 | 4 | 10 | 100 |
| Both | 2 | 4 | 20 | 200 |
| Both | 3 | 6 | 20 | 300 |
| A or B | 1 | 4 | 20 | 300 |
| A or B | 2 | 6 | 20 | 300 |
| A or B | 3 | 6 | 50 | 300 |

Table 2 – Changes in task parameters for different reinforcement blocks.

| Reinforcement block | Selected session | | Selection Rules |
| --- | --- | --- | --- |
| Both | 1 | 1^st^ day at Parameters 1 for Block Both | |
| Both | 2 | 1^st^ day at Parameters 2 for Block Both | |
| Both | 3 | 1^st^ day at Parameters 3 from Block Both | |
| Both | 4 | Last day before change in reinforcement schedule | |
| A or B | 1 | 1st day at Parameters 1 for Block A or B | |
| A or B | 2 | 1^st^ day at Parameters 2 or Block A or B | |
| A or B | 3 | 1^st^ day at Parameters 3 or Block A or B | |
| A or B | 4 | Middle day between selected session 3 and 5 | |
| A or B | 5 | Last day of current reinforcement Block | |

Table 3 – Selected sessions for comparison across animals for all reinforcement blocks.

**
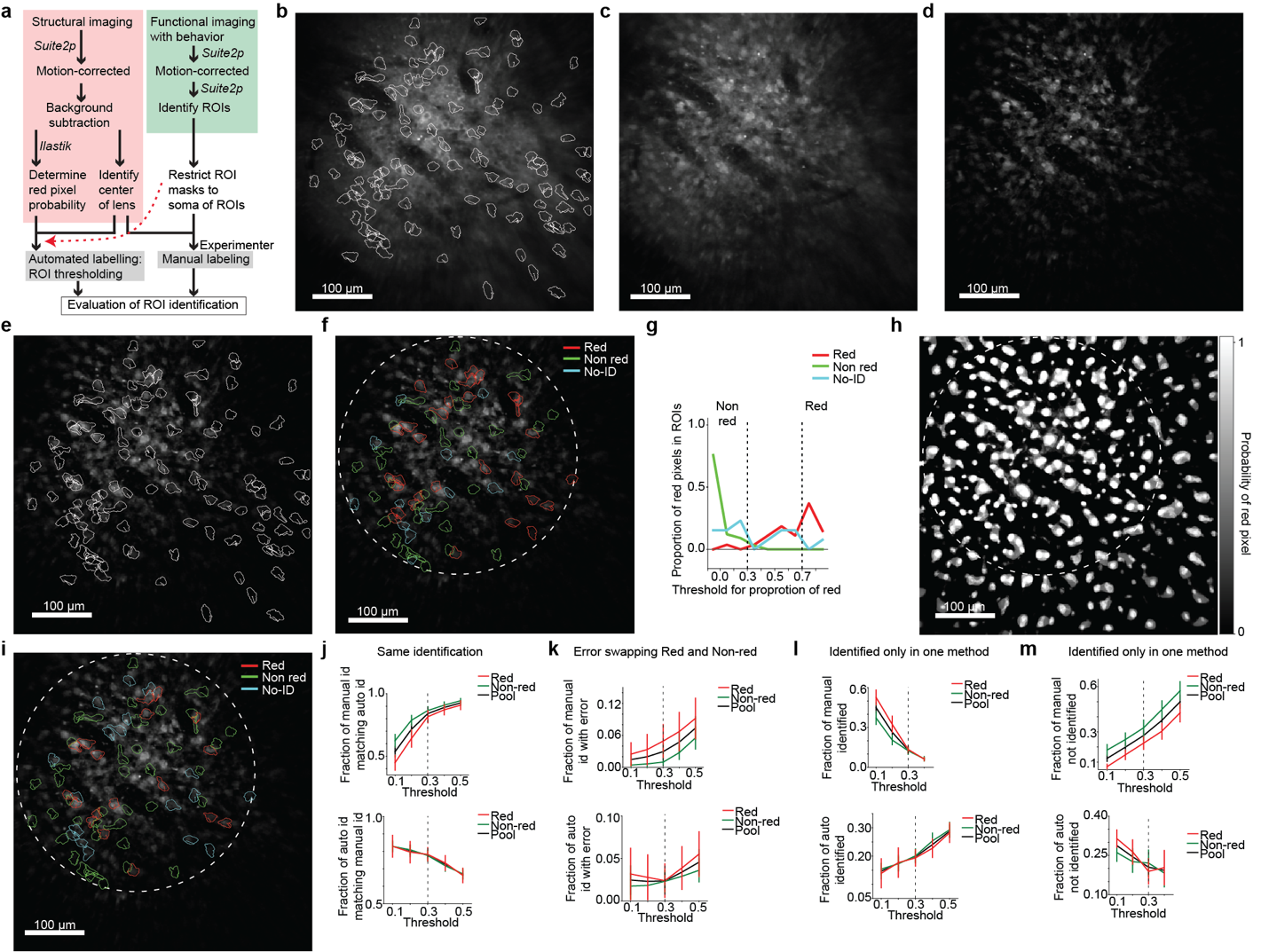
**

**Extended Data Fig. 1 | Method to distinguish neuronal identity based on structural fluorescence.** **a,** Scheme of automated thresholding method to distinguish D1- from D2-MSNs. **b,** Example of Suite2p identified ROIs (in white) overlaid on average image of functional channel (1000 frames of green channel). **c,** Example of average image of structural channel (1000 frames of red channel). **d,** Example of background subtracted structural image (rolling ball of 25 pixels). **e,** Example of ROIs overlaid on structural image used for manual labeling. **f,** Example of manual labeling ROIs within region of interest of GRIN lens (dashed white line): ROIs that experimenter couldn’t confidently label are “No-ID.” **g,** For this example, distribution of red probability in manually labelled groups of ROIs (red for Red, green for no-Red and cyan for no-ID). Dashed lines represent thresholds used for automated method results shown in panel i. **h,** Example of probability of pixel being red determined using machine learning software Ilastik. **i,** Example of automated labeling ROIs within region of interest of GRIN lens (dashed white line). ROIs that automated method couldn’t confidently label with thresholds are “No-ID.” **j-m,** Neuron identification accuracy as a function of automatic labeling threshold. ROIs with proportion of red pixels below threshold are labeled green, and ROIs with proportion of red pixels above 1-threshold are labeled red. **j,** Identification accuracy across thresholds. Top: fraction of ROIs identified manually that were also identified automatically. Bottom: fraction of ROIs identified automatically that were also identified manually. **k,** Errors consisting of swapping “Red” and “Non-red” identity between identification methods. Top: the fraction of manually identified ROIs that were automatically identified with the opposite ID. Bottom: the fraction of automatically identified ROIs that were manually identified with the opposite ID.

**l,** Top: the fraction of manually identified ROIs that were not automatically identified. Bottom: the fraction of automatically identified ROIs that were not manually identified. **m,**  Top: the fraction of manually not-identified ROIs that were automatically identified. Bottom: the fraction automatically not-identified ROIs that were manually identified. Scale bar: 100 μm.

**
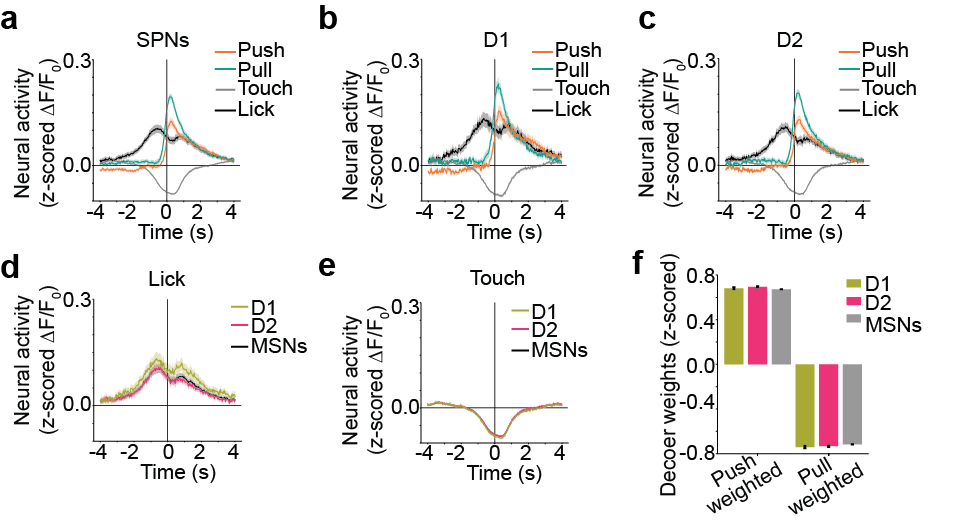
**

**Extended Data Fig. 2 | Neural activity and decoder weights supplementing Figure 2.** **a,** Neural activity (z-scored ΔF/F_0_) averaged across all MSNs for each action and averaged across sessions. Push and pull are 3g cross actions, and activity is locked to force peak. For touch and lick, activity is locked to the middle of the action interval. **b,** Same as “a” but for D1-MSNs. **c,** Same as “a” but for D2-MSNs. **d,** For lick, neural activity (z-scored ΔF/F_0_) averaged across MSNs, D1-MSNs, and D2-MSNs. **e,** Same as “d” but for touch. **a-e,** Data are mean+s.e.m. across n=86 sessions from 8 mice. **f,** Average decoder weights of action-specific ensembles for each cell type. Decoder weights were not significantly different across all cell types for push weighted neurons and pull weighted neurons (see Extended Data Table 2, table ref. 2.1, 2.2, 2.3, 2.4, 2.5, 2.6). Push weighted bar plots are mean+s.e.m. across 63 sessions across 8 mice. Pull weighted bar plots are mean+s.e.m. across 73 sessions across 8 mice.

**
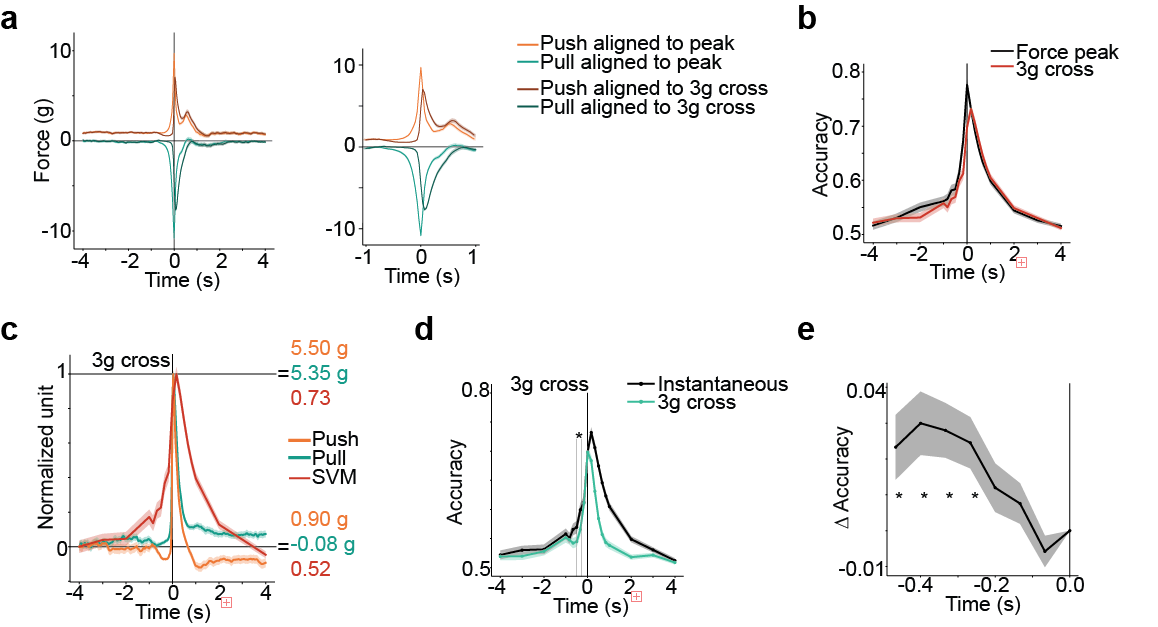
**

**Extended Data Fig. 3 | Action preparation in striatum, supplementing Figure 3**. **a,** Force traces for 3g cross actions with no action in the preceding 0.5 seconds, averaged over trials. Comparison of force locked to peak force (light colors) and 3g cross (dark colors). **b,** Decoding MSN activity to predict action identity with a model fit at each time lag relative to force peak (black) and 3g cross (red). **c,** Decoding MSN activity locked to 3g cross, overlayed on action force (normalized to -4s and 3g cross at 0s). **d,** Accuracy comparison of decoder optimized at each time point before 3g cross (“Instantaneous”) to the decoder optimized at 3g cross. A zoom in is shown in Fig. 3a. **e,** The difference in accuracy between decoders shown in Fig.3c. **a-e,** Data are mean+s.e.m. 86 sessions over 8 mice.

**
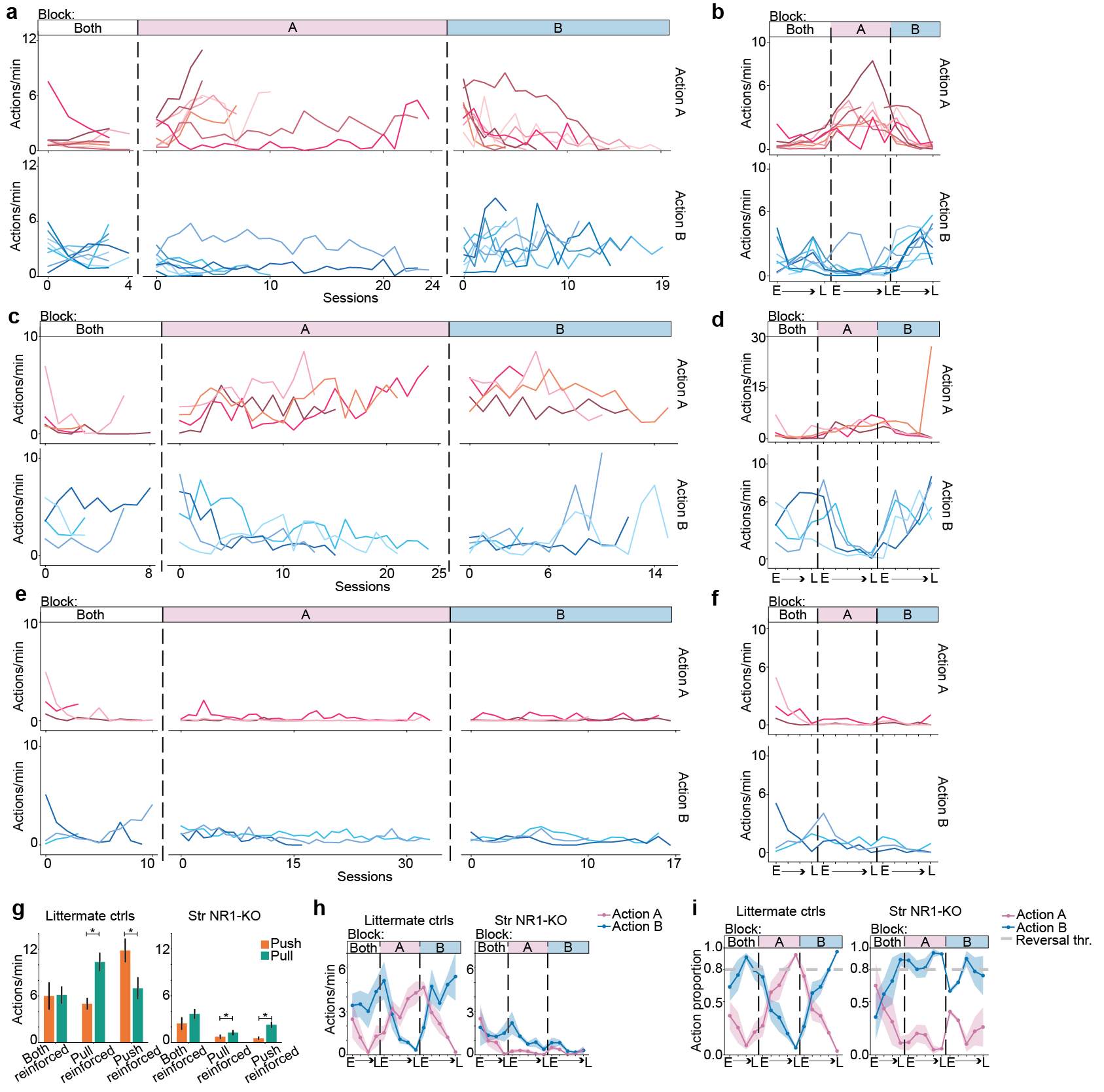
**

**Extended Data Fig. 4 | Supplemental behavior data.** **a,** Rate of task actions for each individual mouse and session in Fig. 2-4 (n=8 mice). **b,** Same as “a” but for selected sessions. **c,** Same as “a” but for each littermate control mouse of the Striatal NR1-KO cohort in Fig. 4 (n=4 mice). **d,** Same as “c” but for selected sessions. **e,** Same as “a” but for each mutant mouse of the Striatal NR1-KO cohort in Fig. 4 (n=3 mice). **f,** Same as “e” but for selected sessions. **g,** Rate of 6g cross actions. Same as Fig. 1d but for striatal NR1-KO and littermate control mice. Data are mean+s.e.m. from n=4 mice (littermate control group) and n=3 mice (NR1-KO group), averaging over sessions in each block (Methods). Littermate controls’ rate for each action was not significantly different when both actions were reinforced (see Extended Data Table 2, table ref. 4.1). Littermate controls performed pull more than push when pull action was reinforced and more push than pull when push action was reinforced (see Extended Data Table 2, table ref. 4.2 and 4.3). NR1-KO mice’ rate for each action was not significantly different when both actions were reinforced (see Extended Data Table 2, table ref. 4.4). NR1-KO mice performed pull more than push when pull action was reinforced and also when push was reinforced (see Extended Data Table 2, table ref. 4.5 and 4.6). **h,** Rate of task actions. Same as Fig. 4b except for NR1-KO and control mice. **i,** Proportion of task actions. Same as Fig. 4c except for NR1-KO and control mice. **h-i,** Data are mean+s.e.m. over n=4 littermate control mice and n=3 NR1-KO for each session.

**
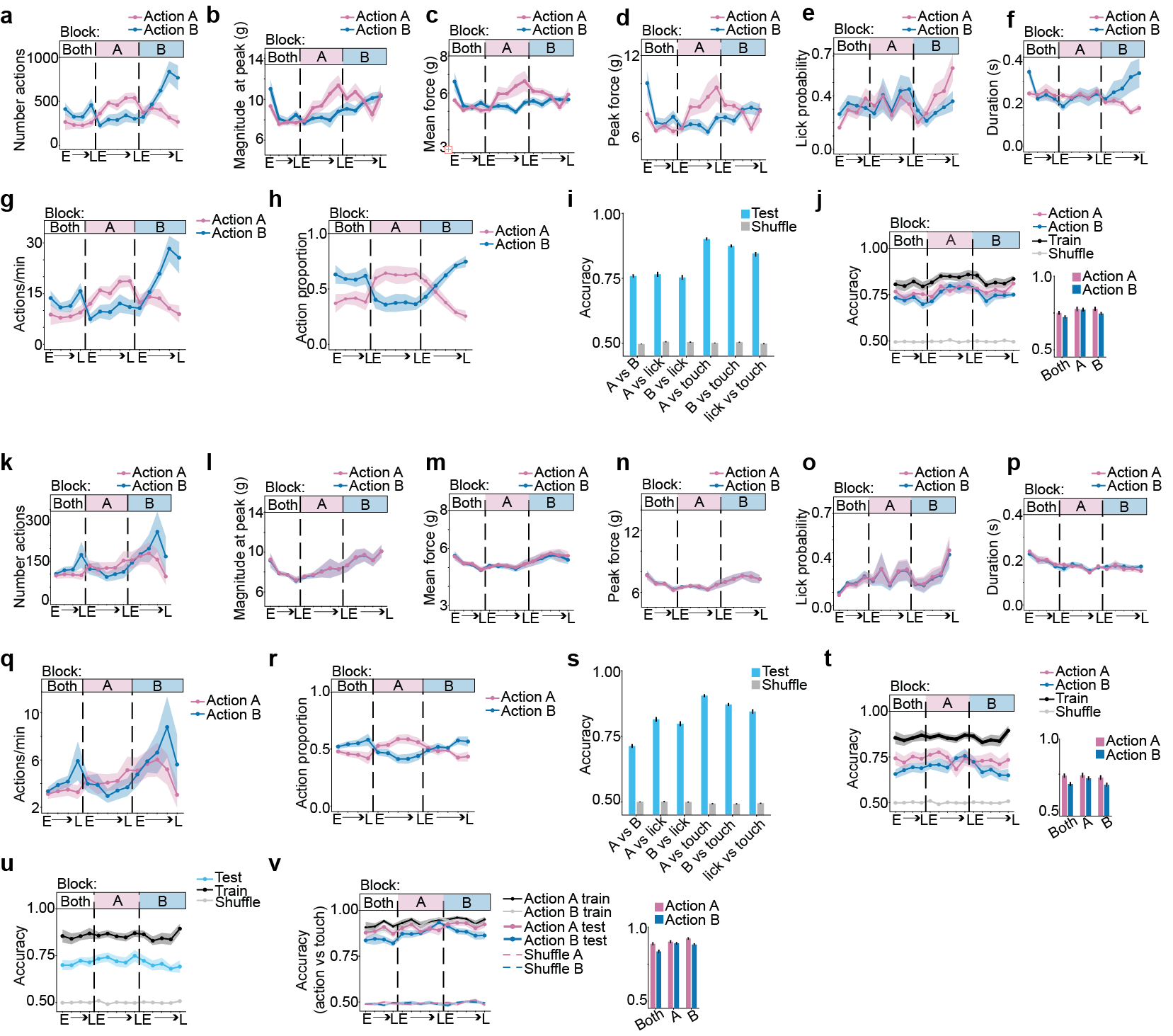
**

**Extended Data Fig. 5 | Striatum encodes isometric forelimb actions in 3g cross trials and trials matched across actions. a-j,** Analysis of 3g cross actions. Data are mean+s.e.m. for n=8 mice for each session, or for each block averaging over sessions. **a,** Number of actions per session. **b,** Magnitude of two-dimensional force vector at time of peak force (in push/pull axis). **c,** Force (in push/pull axis) averaged over the duration of 3g cross actions. **d,** Peak force for 3g cross actions. **e,** Lick probability averaged over duration of 3g cross actions. **f,** Action duration of 3g cross actions across reinforcement blocks. **g,** Rate of 3g cross actions across reinforcement blocks. **h,** Proportion of 3g cross actions with a specific identity (A or B). **i,** Decoding MSN activity at force peak for 3g cross action A and B, predicting action identity between pairs of actions including A, B, touch, and licking. **j,** Decoding MSNs activity at force peak to predict action A or B on single trials. Inset: quantification of accuracy across blocks (average of all sessions in each block). **k-t,** Same as “a-j” but for matched 3g cross actions (Methods). **u,** Decoding MSN activity at force peak to predict action identity. Same as Fig. 4d but for matched 3g cross actions. **v,** Decoding MSN activity at force peak to predict action A from touch and Action B from touch. Same as Fig. 4f but for matched 3g cross actions. Right: average of sessions in each block.

**
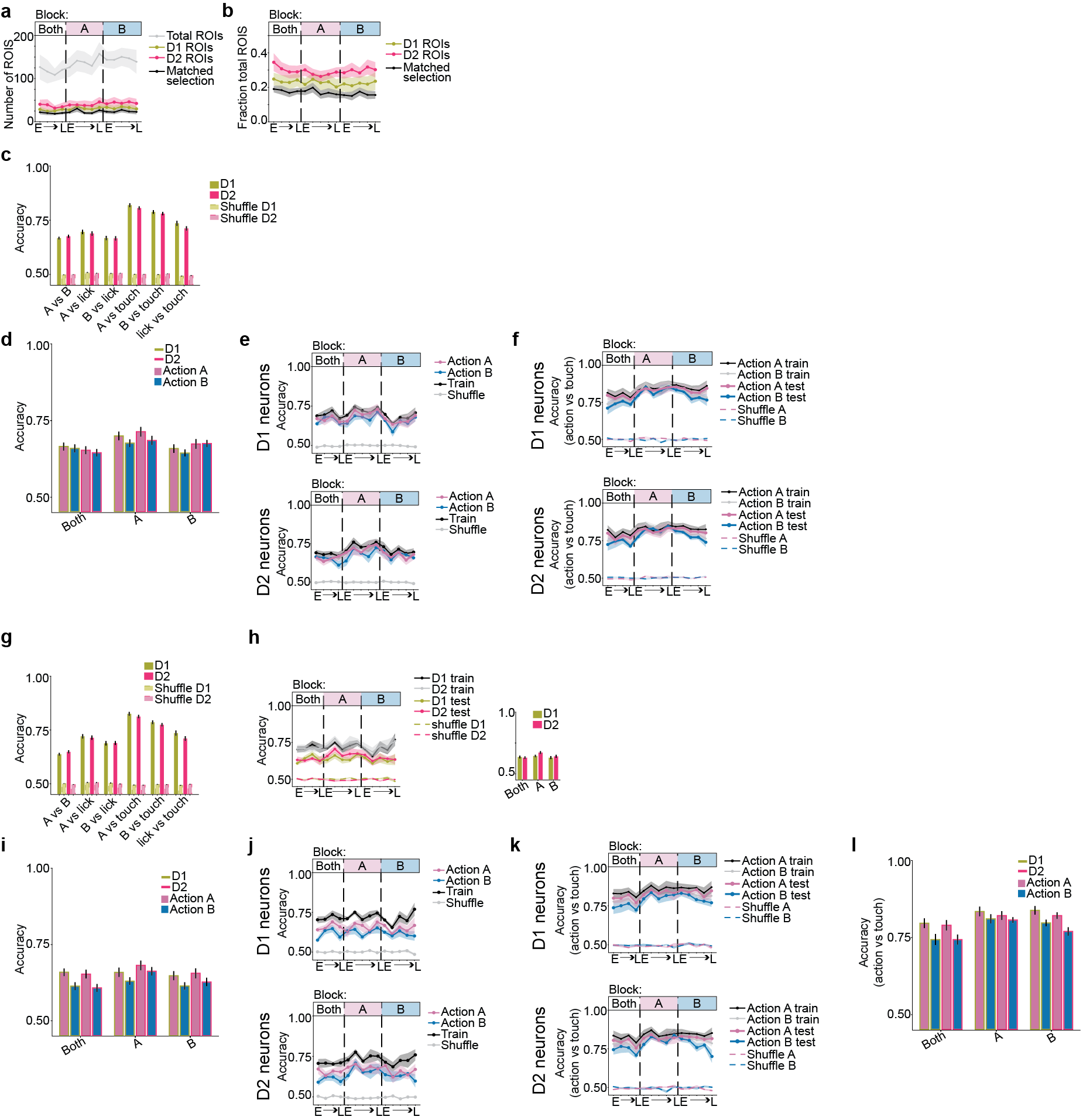
Extended Data Fig. 6 | D1- and D2-MSNs encode isometric forelimb actions in 3g cross trials and trials matched across actions.** **a,** Number of functional ROIs identified with Suite2p. Decoders using D1- and D2-MSNs used ROIs with numbers matched across cell type (shown in black). **b,** Fraction of total ROIs that are D1-MSNs, D2-MSNs, and the number of ROIs matched across D1 and D2. **c-f,** Analysis of 3g cross actions. **c,** Decoding MSN activity at force peak for 3g cross action A and B, predicting action identity between pairs of actions including A, B, touch, and licking. Same as Fig. 2i but analyzing action A and B rather than push and pull. **d,** Accuracy of decoding D1- and D2-MSNs activity at force peak to predict action A versus B, split by action (average of all sessions in each block). **e,** Decoding D1-MSNs (top) and D2-MSNs (bottom) activity at force peak to predict action A or B. **f,** Decoding D1-MSNs (top) and D2-MSNs (bottom) activity at force peak to predict action A versus touch, and action B versus touch. **g-l,** Analysis of matched 3g cross actions. **g,** Same as “c” but for matched 3g cross actions. **h,** Same as Fig. 4e but for matched 3g cross actions. Right: average of sessions in each block. **i-k,** Same as “d-g” but for matched 3g cross actions. **l,** Decoding D1- and D2-MSN activity at force peak to predict action A from touch and Action B from touch, averaging sessions in each block. Same as Fig. 4g but for matched 3g cross actions. **a-l,** Data are mean+s.e.m. for n=8 mice for each session, or for each block averaging over sessions.


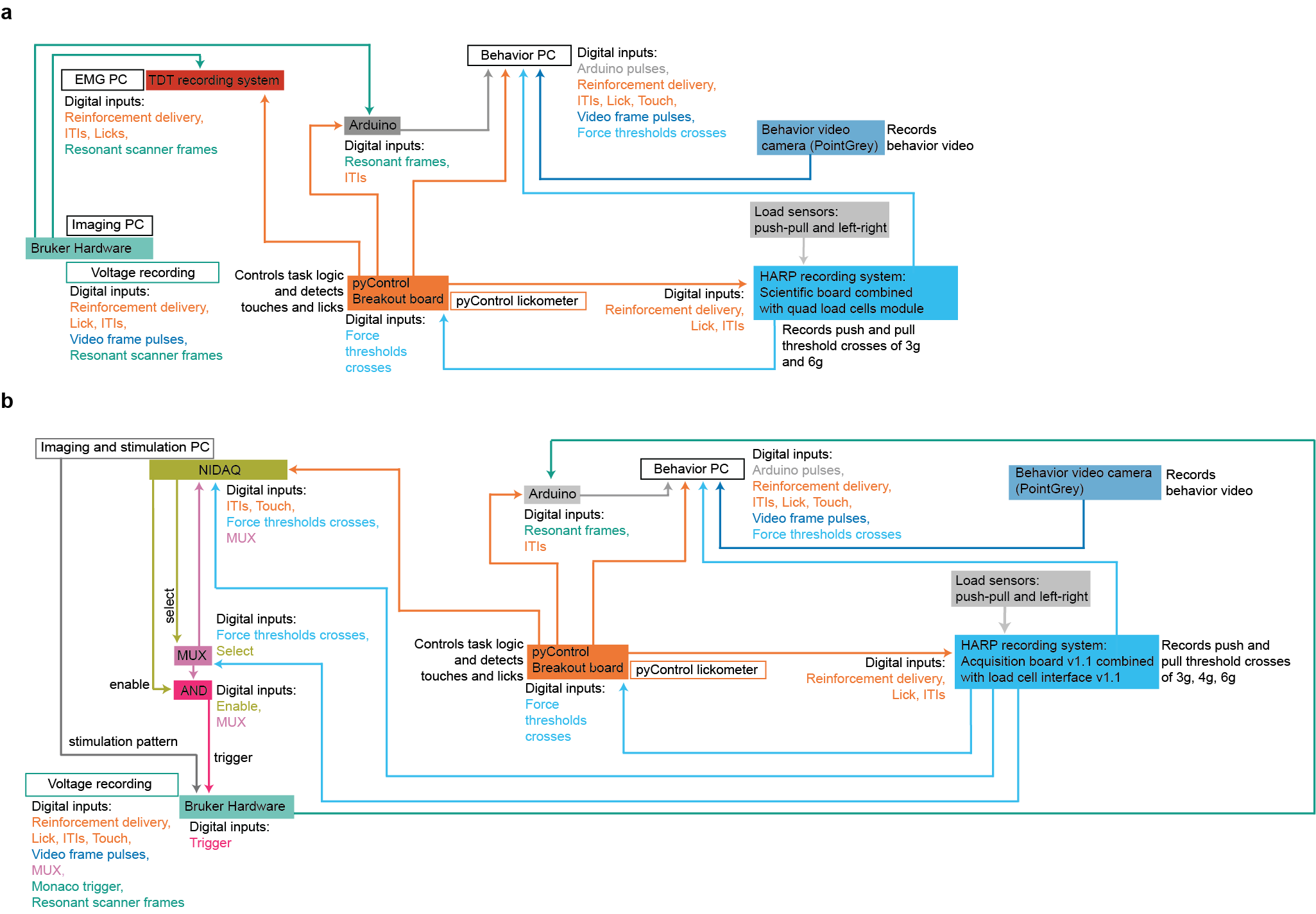


**Extended Data Fig. 7 | Experiment hardware setup schematics.** **a,** Schematic of hardware setup to run the two-action isometric task. **b,** Schematic of hardware setup to run the adaptive isometric task and closed-loop holographic stimulation experiments.
