# Supplementary material for: Striatal ensembles specify and control granular forelimb actions
Source: bioRxiv. 2025 Dec 8:2025.12.03.692128. Preprint. [Version 1] doi: 10.64898/2025.12.03.692128 (PMC12713657; doi:10.64898/2025.12.03.692128)
Supplement: Supplement 2 [file media-2.docx]

Extended Data Table 1 – Summary of statistical tests in Figures

| Table ref. | Figure Panel | Group | Statistical test | Sample size | Test Statistic | *P* value | Sig. | Notes |
| --- | --- | --- | --- | --- | --- | --- | --- | --- |
| 1.1 | 1.d | Action rate in Both reinforced block: push vs pull | Paired t-test | 32 sessions (4 sessions/mouse, 8 mice) | 2.56 | 1.6e-2 | * | All sessions within block |
| 1.2 | 1.d | Action rate at Pull reinforced block: push vs pull | Paired t-test | 40 sessions (5 sessions/mouse, 8 mice) | 6.27 | 2.43e-7 | * | All sessions within block |
| 1.3 | 1.d | Action rate at Push reinforced block: push vs pull | Paired t-test | 40 sessions (5 sessions/mouse, 8 mice) | 3.38 | 1.65e-3 | * | All sessions within block |
| 1.4 | 1.f | Action onset vs baseline: biceps | Paired t-test | 41 sessions (4 mice) | 5.47 | 2.61e-6 | * | 100 ms at onset vs 100 ms 1 s before onset |
| 1.5 | 1.f | Action onset vs baseline: triceps | Paired t-test | 41 sessions (4 mice) | 7.84 | 1.31e-9 | * | 100 ms at onset vs 100 ms 1 s before onset |
| 1.6 | 1.f | Action onset vs baseline: PL | Paired t-test | 41 sessions (4 mice) | 4.64 | 3.73e-5 | * | 100 ms at onset vs 100 ms 1 s before onset |
| 1.7 | 1.f | Action onset vs baseline: EDC | Paired t-test | 41 sessions (4 mice) | 3.68 | 6.8e-4 | * | 100 ms at onset vs 100 ms 1 s before onset |
| 1.8 | 1.g | Action onset vs baseline: biceps | Paired t-test | 41 sessions (4 mice) | 12.02 | 7.41e-15 | * | 100 ms at onset vs 100 ms 1 s before onset |
| 1.9 | 1.g | Action onset vs baseline: triceps | Paired t-test | 41 sessions (4 mice) | 6.76 | 4.11e-8 | * | 100 ms at onset vs 100 ms 1 s before onset |
| 1.10 | 1.g | Action onset vs baseline: PL | Paired t-test | 41 sessions (4 mice) | 7.39 | 5.37e-9 | * | 100 ms at onset vs 100 ms 1 s before onset |
| 1.11 | 1.g | Action onset vs baseline: EDC | Paired t-test | 41 sessions (4 mice) | 7.26 | 8.14e-9 | * | 100 ms at onset vs 100 ms 1 s before onset |
| 1.12 | 1.h | Action onset vs baseline: PC1 push | Paired t-test | 41 sessions (4 mice) | 6.66 | 5.61e-8 | * | 100 ms at onset vs 100 ms 1 s before onset |
| 1.13 | 1.h | Action onset vs baseline: PC1 pull | Paired t-test | 41 sessions (4 mice) | 13.90 | 6.6e-17 | * | 100 ms at onset vs 100 ms 1 s before onset |
| 1.14 | 1.i | Average vs differential modulation across push and pull | Paired t-test | 41 sessions (4 mice) | 4.97 | 1.29e-5 | * | 100 ms centered at onset |
| 1.15 | 1.j | Difference across vs within action: push | Paired t-test | 41 sessions (4 mice) | 4.50 | 5.76e-5 | * | 100 ms centered at onset |
| 1.16 | 1.j | Difference across vs within action: pull | Paired t-test | 41 sessions (4 mice) | 6.60 | 6.87e-8 | * | 100 ms centered at onset |
| 2.1 | 2.c, top - push | Difference between baseline vs force peak: neural activity D1 | Paired t-test | 86 sessions (8 mice) | -9.19 | 2.06e-14 | * | ~90 ms (3 frames) at force peak and -0.5 s before peak |
| 2.2 | 2.c, top - push | Difference between baseline vs force peak: neural activity D2 | Paired t-test | 86 sessions (8 mice) | 11.61 | 2.01e-14 | * | ~90 ms (3 frames) at force peak and -0.5 s before peak |
| 2.3 | 2.c, top - pull | Difference between baseline vs force peak: neural activity D1 | Paired t-test | 86 sessions (8 mice) | -14.36 | 1.45e-24 | * | ~90 ms (3 frames) at force peak and -0.5 s before peak |
| 2.4 | 2.c, top - pull | Difference between baseline vs force peak: neural activity D2 | Paired t-test | 86 sessions (8 mice) | -14.78 | 2.51e-25 | * | ~90 ms (3 frames) at force peak and -0.5 s before peak |
| 2.5 | 2.c, bottom right | Difference between D1 and D2 at push peak | Paired t-test | 86 sessions (8 mice) | 2.15 | 0.03 | * | -0.5 s to 0.5 s at force peak |
| 2.6 | 2.c, bottom right | Difference between D1 and D2 at pull peak | Paired t-test | 86 sessions (8 mice) | 2.49 | 0.01 | * | -0.5 s to 0.5 s at force peak |
| 2.7 | 2.e | Accuracy prediction at force peak: data vs shuffle | Paired t-test | 87 sessions (8 mice) | 25.85 | 2.55e-42 | * | 5 frames centered at peak force |
| 2.8 | 2.e | Accuracy prediction at force peak: data vs shuffle | Paired t-test | 87 sessions (8 mice) | 11.45 | 5.50e-19 | * | 4 sec before force peak |
| 2.9 | 2.g | Accuracy prediction at force peak: data vs shuffle | Paired t-test | 87 sessions (8 mice) | 12.68 | 2.20e-21 | * | 0.5 sec before force peak |
| 2.10 | 2.g | Accuracy prediction at force peak: D1 data vs shuffle | Paired t-test | 87 sessions (8 mice) | 16.99 | 3.17e-29 | * | 5 frames centered at peak force |
| 2.11 | 2.g | Accuracy prediction at force peak: D2 data vs shuffle | Paired t-test | 87 sessions (8 mice) | 16.24 | 6.18e-28 | * | 5 frames centered at peak force |
| 2.12 | 2.g | Accuracy prediction at force peak: D2 data vs D1 data | Paired t-test | 87 sessions (8 mice) | -0.05 | 0.96 | n.s. | 5 frames centered at peak force |
| 2.13 | 2.g | Accuracy prediction at force peak: D1 data vs shuffle | Paired t-test | 87 sessions (8 mice) | 8.91 | 7.49e-14 | * | 4 sec before force peak |
| 2.14 | 2.g | Accuracy prediction at force peak: D2 data vs shuffle | Paired t-test | 87 sessions (8 mice) | 7.94 | 6.89e-12 | * | 4 sec before force peak |
| 2.15 | 2.g | Accuracy prediction at force peak: D1 data vs shuffle | Paired t-test | 87 sessions (8 mice) | 10.33 | 9.72e-17 | * | 0.5 sec before force peak |
| 2.16 | 2.g | Accuracy prediction at force peak: D2 data vs shuffle | Paired t-test | 87 sessions (8 mice) | 8.52 | 4.57e-13 | * | 0.5 sec before force peak |
| 2.17 | 2.h | Accuracy prediction: push vs pull | Paired t-test | 86 sessions (8 mice) | 36.44 | 3.82e-62 | * |  |
| 2.18 | 2.h | Accuracy prediction: push vs lick | Paired t-test | 86 sessions (8 mice) | 26.29 | 1.21e-45 | * |  |
| 2.19 | 2.h | Accuracy prediction: pull vs lick | Paired t-test | 86 sessions (8 mice) | 28.99 | 2.81e-49 | * |  |
| 2.20 | 2.h | Accuracy prediction: push vs touch | Paired t-test | 86 sessions (8 mice) | 54.20 | 1.24e-79 | * |  |
| 2.21 | 2.h | Accuracy prediction: pull vs touch | Paired t-test | 86 sessions (8 mice) | 79.37 | 6.43e-97 | * |  |
| 2.22 | 2.h | Accuracy prediction: lick vs touch | Paired t-test | 86 sessions (8 mice) | 36.38 | 5.90e-58 | * |  |
| 2.23 | 2.i | Accuracy predicting push vs pull: D1 vs D2 | Paired t-test | 86 sessions (8 mice) | -1.44 | 0.15 | n.s. |  |
| 2.24 | 2.i | D1 - Accuracy prediction: push vs pull | Paired t-test | 86 sessions (8 mice) | 24.38 | 6.49e-41 | * |  |
| 2.25 | 2.i | D1 - Accuracy prediction: push vs lick | Paired t-test | 86 sessions (8 mice) | 15.39 | 2.41e-25 | * |  |
| 2.26 | 2.i | D1 - Accuracy prediction: pull vs lick | Paired t-test | 86 sessions (8 mice) | 19.72 | 4.83e-32 | * |  |
| 2.27 | 2.i | D1 - Accuracy prediction: push vs touch | Paired t-test | 86 sessions (8 mice) | 28.51 | 3.16e-46 | * |  |
| 2.28 | 2.i | D1 - Accuracy prediction: pull vs touch | Paired t-test | 86 sessions (8 mice) | 38.42 | 6.89e-58 | * |  |
| 2.29 | 2.i | D1 - Accuracy prediction: lick vs touch | Paired t-test | 86 sessions (8 mice) | 19.14 | 3.35e-31 | * |  |
| 2.30 | 2.i | D2 - Accuracy prediction: push vs pull | Paired t-test | 86 sessions (8 mice) | 22.27 | 6.38e-38 | * |  |
| 2.31 | 2.i | D2 - Accuracy prediction: push vs lick | Paired t-test | 86 sessions (8 mice) | 16.28 | 8.32e-27 | * |  |
| 2.32 | 2.i | D2 - Accuracy prediction: pull vs lick | Paired t-test | 86 sessions (8 mice) | 18.47 | 3.24e-30 | * |  |
| 2.33 | 2.i | D2 - Accuracy prediction: push vs touch | Paired t-test | 86 sessions (8 mice) | 34.04 | 1.94e-52 | * |  |
| 2.34 | 2.i | D2 - Accuracy prediction: pull vs touch | Paired t-test | 86 sessions (8 mice) | 37.93 | 2.60e-56 | * |  |
| 2.35 | 2.i | D2 - Accuracy prediction: lick vs touch | Paired t-test | 86 sessions (8 mice) | 19.83 | 3.37e-32 | * |  |
| 2.36 | 2.j | Force peak SVM projection vs baseline: push | Paired t-test | 86 sessions (8 mice) | -10.75 | 1.36e-17 | * | -0.5 s to peak and at force peak |
| 2.37 | 2.j | Force peak SVM projection vs baseline: pull | Paired t-test | 86 sessions (8 mice) | 17.05 | 2.57e-29 | * | -0.5 s to peak and at force peak |
| 2.38 | 2.j | Force peak SVM projection pus vs pull | Paired t-test | 86 sessions (8 mice) | 25.33 | 1.21e-41 | * | -0.5 s to peak and at force peak |
| 2.39 | 2.k | Neural activity at peak vs before: push | Paired t-test | 86 sessions (8 mice) | -15.32 | 2.67e-26 | * | -0.5 s to peak and at force peak |
| 2.40 | 2.k | Push neural activity at peak vs pull neural activity at peak | Paired t-test | 86 sessions (8 mice) | 19.33 | 4.70e-33 | * | -0.5 s to peak and at force peak |
| 2.41 | 2.l | Neural activity at peak vs before: pull | Paired t-test | 86 sessions (8 mice) | -21.60 | 1.71e-36 | * | -0.5 s to peak and at force peak |
| 2.42 | 2.l | Pull neural activity at peak vs push neural activity at peak | Paired t-test | 86 sessions (8 mice) | -21.21 | 6.45e-36 | * | -0.5 s to peak and at force peak |
| 2.43 | 2.m | Neural activity at peak D1 vs D2 | Paired t-test | 63 sessions (8 mice) | 0.82 | 0.41 | n.s. | -0.5 s to peak and at force peak |
| 2.44 | 2.n | Neural activity at peak D1 vs D2 | Paired t-test | 73 sessions (8 mice) | 0.68 | 0.50 | n.s. | -0.5 s to peak and at force peak |
| 3.1 | 3.a | Accuracy prediction at 3g cross: data vs shuffle | Paired t-test | 84 sessions (8 mice) | 13.08 | 3.78e-22 | * | Average from -0.5 s to 0 s of 3g cross |
| 3.2 | 3.a | Accuracy prediction vs force: push action | Paired t-test | 84 sessions (8 mice) | 4.13 | 8.24e-5 | * | Average from -0.5 s to 0 s of 3g cross |
| 3.3 | 3.a | Accuracy prediction vs force: pull action | Paired t-test | 84 sessions (8 mice) | 3.53 | 6.61e-4 | * | Average from -0.5 s to 0 s of 3g cross |
| 3.4 | 3.b | Angle alignment vs time | Linear regression | 86 sessions (8 mice) | -0.722 | 3.15e-113 | * |  |
| 3.5 | 3.b | Angle alignment at force vs 3g cross: -0.467 s | Paired t-test | 86 sessions (8 mice) | 34.50 | 3.79e-52 | * | From selected time window to 0 s of 3g cross |
| 3.6 | 3.b | Angle alignment at force vs 3g cross: -0.4 s | Paired t-test | 86 sessions (8 mice) | 31.22 | 1.08e-48 | * | From selected time window to 0 s of 3g cross |
| 3.7 | 3.b | Angle alignment at force vs 3g cross: -0.33 s | Paired t-test | 86 sessions (8 mice) | 30.30 | 1.16e-47 | * | From selected time window to 0 s of 3g cross |
| 3.8 | 3.b | Angle alignment at force vs 3g cross: -0.267 s | Paired t-test | 86 sessions (8 mice) | 29.07 | 3.0e-46 | * | From selected time window to 0 s of 3g cross |
| 3.9 | 3.b | Angle alignment at force vs 3g cross: -0.2 s | Paired t-test | 86 sessions (8 mice) | 29.61 | 7.06e-47 | * | From selected time window to 0 s of 3g cross |
| 3.10 | 3.b | Angle alignment at force vs 3g cross: -0.133 s | Paired t-test | 86 sessions (8 mice) | 25.89 | 2.25e-42 | * | From selected time window to 0 s of 3g cross |
| 3.11 | 3.b | Angle alignment at force vs 3g cross: -0.067 s | Paired t-test | 86 sessions (8 mice) | 25.81 | 2.91e-42 | * | From selected time window to 0 s of 3g cross |
| 3.12 | 3.c | Accuracy of decoder: instantaneous vs 3g cross optimized | Paired t-test | 86 sessions (8 mice) | 3.25 | 1.65e-3 | * | Average from -0.5 s to 0 s of 3g cross |
| 3.13 | 3.c | Angle alignment at force vs 3g cross: -0.467 s | Paired t-test | 86 sessions (8 mice) | 2.57 | 1.20e-2 | * | From selected time window to 0 s of 3g cross |
| 3.14 | 3.c | Angle alignment at force vs 3g cross: -0.4 s | Paired t-test | 86 sessions (8 mice) | 3.37 | 1.11e-3 | * | From selected time window to 0 s of 3g cross |
| 3.15 | 3.c | Angle alignment at force vs 3g cross: -0.33 s | Paired t-test | 86 sessions (8 mice) | 3.65 | 4.46e-4 | * | From selected time window to 0 s of 3g cross |
| 3.16 | 3.c | Angle alignment at force vs 3g cross: -0.267 s | Paired t-test | 86 sessions (8 mice) | 3.41 | 9.83e-4 | * | From selected time window to 0 s of 3g cross |
| 3.17 | 3.e | Time of peak SVM projection relative to force peak: 3g cross vs peak | Paired t-test | 86 sessions (8 mice) | 3.03 | 0.003 | * | First point to cross 99^th^ percentile (-1 to 1 s) |
| 3.18 | 3.e | Time of peak SVM projection relative to force peak: late prep. vs peak | Paired t-test | 86 sessions (8 mice) | 3.52 | 7e-4 | * | First point to cross 99^th^ percentile (-1 to 1 s) |
| 3.19 | 3.e | Time of peak SVM projection relative to force peak: early prep. vs peak | Paired t-test | 86 sessions (8 mice) | 4.48 | 2.30e-5 | * | First point to cross 99^th^ percentile (-1 to 1 s) |
| 3.20 | 3.e | Modeled latency as dependent on decoder type | Linear mixed effect model | 86 sessions (8 mice) | Slope: 0.05 | 9.66e-9 | * | Data grouped by session; modeled as random effect |
| 3.21 | 3.f | Time of peak SVM projection relative to force peak: 3g cross vs peak | Paired t-test | 86 sessions (8 mice) | 2.96 | 4.00e-3 | * | First point to cross 99^th^ percentile (-1 to 1 s) |
| 3.22 | 3.f | Time of peak SVM projection relative to force peak: late prep. vs peak | Paired t-test | 86 sessions (8 mice) | 6.46 | 5.98e-9 | * | First point to cross 99^th^ percentile (-1 to 1 s) |
| 3.23 | 3.f | Time of peak SVM projection relative to force peak: early prep. vs peak | Paired t-test | 86 sessions (8 mice) | 9.20 | 1.97e-14 | * | First point to cross 99^th^ percentile (-1 to 1 s) |
| 3.24 | 3.f | Modeled latency as dependent on decoder type | Linear mixed effect model | 86 sessions (8 mice) | Slope: 0.08 | 6.83e-28 | * | Data grouped by session; modeled as random effect |
| 3.25 | 3.h | Time of peak SVM projection relative to force peak: 3g cross vs peak | Paired t-test | 86 sessions (8 mice) | 2.65 | 9.51e-3 | * |  |
| 3.26 | 3.h | Time of peak SVM projection relative to force peak: late prep. vs peak | Paired t-test | 86 sessions (8 mice) | 3.33 | 1.28e-3 | * |  |
| 3.27 | 3.h | Time of peak SVM projection relative to force peak: early prep. vs peak | Paired t-test | 86 sessions (8 mice) | 3.20 | 1.91e-3 | * |  |
| 3.28 | 3.h | Modeled latency as dependent on decoder type | Linear mixed effect model | 86 sessions (8 mice) | Slope: 0.04 | 1.04e-5 | * | Data grouped by session; modeled as random effect |
| 3.29 | 3.i | Time of peak SVM projection relative to force peak: 3g cross vs peak | Paired t-test | 67 sessions (8 mice) | 4.15 | 9.72e-5 | * |  |
| 3.30 | 3.i | Time of peak SVM projection relative to force peak: late prep. vs peak | Paired t-test | 67 sessions (8 mice) | 2.86 | 5.6e-3 | * |  |
| 3.31 | 3.i | Time of peak SVM projection relative to force peak: early prep. vs peak | Paired t-test | 67 sessions (8 mice) | 3.82 | 3.0e-4 | * |  |
| 3.32 | 3.i | Modeled latency as dependent on decoder type | Linear mixed effect model | 67 sessions (8 mice) | Slope: 0.04 | 3.9e-5 | * | Data grouped by session; modeled as random effect |
| 3.33 | 3.j | Time of peak SVM projection relative to force peak: 3g cross vs peak | Paired t-test | 78 sessions (8 mice) | 2.41 | 1.9e-2 | * |  |
| 3.34 | 3.j | Time of peak SVM projection relative to force peak: late prep. vs peak | Paired t-test | 78 sessions (8 mice) | 2.77 | 7.1e-3 | * |  |
| 3.35 | 3.j | Time of peak SVM projection relative to force peak: early prep. vs peak | Paired t-test | 78 sessions (8 mice) | 3.45 | 9.3e-4 | * |  |
| 3.36 | 3.j | Modeled latency as dependent on decoder type | Linear mixed effect model | 78 sessions (8 mice) | Slope: 0.07 | 6.86e-5 | * | Data grouped by session; modeled as random effect |
| 3.37 | 3.k | Time of peak SVM projection relative to force peak: 3g cross vs peak | Paired t-test | 86 sessions (8 mice) | 4.34 | 3.91e-5 | * |  |
| 3.38 | 3.k | Time of peak SVM projection relative to force peak: late prep. vs peak | Paired t-test | 86 sessions (8 mice) | 4.29 | 4.73e-5 | * |  |
| 3.39 | 3.k | Time of peak SVM  projection relative to force peak: early prep. vs peak | Paired t-test | 86 sessions (8 mice) | 5.37 | 6.49e-7 | * |  |
| 3.40 | 3.k | Modeled latency as dependent on decoder type | Linear mixed effect model | 86 sessions (8 mice) | Slope: 0.04 | 3.07e-11 | * | Data grouped by session; modeled as random effect |
| 3.41 | 3.l | Time of peak SVM projection relative to force peak: 3g cross vs peak | Paired t-test | 71 sessions (8 mice) | 0.87 | 0.39 | n.s. |  |
| 3.42 | 3.l | Time of peak SVM projection relative to force peak: late prep. vs peak | Paired t-test | 71 sessions (8 mice) | 2.56 | 1.28e-2 | * |  |
| 3.43 | 3.l | Time of peak SVM projection relative to force peak: early prep. vs peak | Paired t-test | 71 sessions (8 mice) | 6.01 | 7.72e-8 | * |  |
| 3.44 | 3.l | Modeled latency as dependent on decoder type | Linear mixed effect model | 71 sessions (8 mice) | Slope: 0.05 | 4.67e-11 | * | Data grouped by session; modeled as random effect |
| 3.45 | 3.m | Time of peak SVM projection relative to force peak: 3g cross vs peak | Paired t-test | 84 sessions (8 mice) | 4.55 | 1.79e-5 | * |  |
| 3.46 | 3.m | Time of peak SVM projection relative to force peak: late prep. vs peak | Paired t-test | 84 sessions (8 mice) | 4.10 | 9.79e-5 | * |  |
| 3.47 | 3.m | Time of peak SVM projection relative to force peak: early prep. vs peak | Paired t-test | 84 sessions (8 mice) | 5.14 | 1.88e-6 | * |  |
| 3.48 | 3.m | Modeled latency as dependent on decoder type | Linear mixed effect model | 84 sessions (8 mice) | Slope: 0.06 | 4.67e-11 | * | Data grouped by session; modeled as random effect |
| 4.1 | 4.b | Action rate at Both reinforced: A vs B | Paired t-test | 32 sessions (8 mice) | -3.36 | 2.11e-3 | * |  |
| 4.2 | 4.b | Modeled Rate of action as dependent on session: Block A action A | Linear mixed effect model | 40 sessions (8 mice) | Slope: 0.74 | 2.6e-5 | * | Data grouped by animal; modeled as random effect |
| 4.3 | 4.b | Modeled Rate of action as dependent on session: Block A action B | Linear mixed effect model | 40 sessions (8 mice) | Slope: -0.29 | 2.1e-3 | * | Data grouped by animal; modeled as random effect Negative slope indicates drops with sessions |
| 4.4 | 4.b | Modeled Rate of action as dependent on session: Block B action A | Linear mixed effect model | 40 sessions (8 mice) | Slope: -1.14 | 1.2e-14 | * | Data grouped by animal; modeled as random effect  Negative slope indicates drops with sessions |
| 4.5 | 4.b | Modeled Rate of action as dependent on session: Block B action B | Linear mixed effect model | 40 sessions (8 mice) | Slope: 0.59 | 1.2e-14 | * | Data grouped by animal; modeled as random effect |
| 4.6 | 4.c | Modeled Proportion of action as dependent on session: Block A action A | Linear mixed effect model | 40 sessions (8 mice) | Slope: 0.09 | 4.11e-4 | * | Data grouped by animal; modeled as random effect |
| 4.7 | 4.c | Modeled Proportion of action as dependent on session: Block A action B | Linear mixed effect model | 40 sessions (8 mice) | Slope: -0.09 | 4.1e-4 | * | Data grouped by animal; modeled as random effect Negative slope indicates drops with sessions |
| 4.8 | 4.c | Modeled Proportion of action as dependent on session: Block B action A | Linear mixed effect model | 40 sessions (8 mice) | Slope: -0.16 | 2.0e-17 | * | Data grouped by animal; modeled as random effect  Negative slope indicates drops with sessions |
| 4.9 | 4.c | Modeled Proportion of action as dependent on session: Block B action B | Linear mixed effect model | 40 sessions (8 mice) | Slope: 0.16 | 2.0e-17 | * | Data grouped by animal; modeled as random effect |
| 4.10 | 4.d | 1^st^ session: Accuracy predicting action identity: test vs shuffle | Paired t-test | 8 sessions (8 mice) | 7.40 | 1.5e-4 | * |  |
| 4.11 | 4.d | Block Both: Accuracy predicting action identity: test vs shuffle | Paired t-test | 32 sessions (8 mice) | 18.15 | 9.95e-18 | * | All sessions in block |
| 4.12 | 4.d | Block A: Accuracy predicting action identity: test vs shuffle | Paired t-test | 40 sessions (8 mice) | 21.50 | 3.92e-22 | * | All sessions in block |
| 4.13 | 4.d | Block B: Accuracy predicting action identity: test vs shuffle | Paired t-test | 40 sessions (8 mice) | 23.90 | 6.73e-25 | * | All sessions in block |
| 4.14 | 4.d | Accuracy predicting action identity: block Both vs Block A | Independent t-test | 72 sessions (8 mice) | 2.18 | 3.29e-2 | * | All sessions in block |
| 4.15 | 4.d | Accuracy predicting action identity: block Both vs Block B | Independent t-test | 72 sessions (8 mice) | 1.64 | 1.05e-1 | n.s. | All sessions in block |
| 4.16 | 4.e | D1- Block Both: Accuracy predicting action identity: test vs shuffle | Paired t-test | 32 sessions (8 mice) | 13.83 | 3.36e-13 | * | All sessions in block |
| 4.17 | 4.e | D1 - Block A: Accuracy predicting action identity: test vs shuffle | Paired t-test | 40 sessions (8 mice) | 15.64 | 5.76e-16 | * | All sessions in block |
| 4.18 | 4.e | D1 - Block B: Accuracy predicting action identity: test vs shuffle | Paired t-test | 40 sessions (8 mice) | 13.28 | 2.48e-14 | * | All sessions in block |
| 4.19 | 4.e | D2- Block Both: Accuracy predicting action identity: test vs shuffle | Paired t-test | 32 sessions (8 mice) | 13.39 | 6.62e-13 | * | All sessions in block |
| 4.20 | 4.e | D2 - Block A: Accuracy predicting action identity: test vs shuffle | Paired t-test | 40 sessions (8 mice) | 13.42 | 3.19e-14 | * | All sessions in block |
| 4.21 | 4.e | D2 - Block B: Accuracy predicting action identity: test vs shuffle | Paired t-test | 40 sessions (8 mice) | 13.65 | 1.18e-14 | * | All sessions in block |
| 4.22 | 4.e; bar plot | Block Both: D1 vs D2 accuracy difference | Paired t-test | 32 sessions (8 mice) | 1.46 | 1.57e-1 | n.s. | All sessions in block |
| 4.23 | 4.e; bar plot | Block A: D1 vs D2 accuracy difference | Paired t-test | 40 sessions (8 mice) | -1.34 | 1.89e-1 | n.s. | All sessions in block |
| 4.24 | 4.e; bar plot | Block B: D1 vs D2 accuracy difference | Paired t-test | 40 sessions (8 mice) | -2.01 | 5.28e-2 | n.s. | All sessions in block |
| 4.25 | 4.f | Action A - 1^st^ session: Accuracy predicting action identity vs touch: test vs shuffle | Paired t-test | 8 sessions (8 mice) | 15.35 | 1.20e-6 | * |  |
| 4.26 | 4.f | Action B - 1^st^ session: Accuracy predicting action identity vs touch: test vs shuffle | Paired t-test | 8 sessions (8 mice) | 10.28 | 1.79e-5 | * |  |
| 4.27 | 4.f | Action A - Block Both: Accuracy predicting action identity vs touch: test vs shuffle | Paired t-test | 32 sessions (8 mice) | 28.27 | 3.51e-23 | * | All sessions in block |
| 4.28 | 4.f | Action A - Block A: Accuracy predicting action identity vs touch: test vs shuffle | Paired t-test | 40 sessions (8 mice) | 44.68 | 3.92e-33 | * | All sessions in block |
| 4.29 | 4.f | Action A - Block B: Accuracy predicting action identity vs touch: test vs shuffle | Paired t-test | 40 sessions (8 mice) | 50.05 | 5.32e-37 | * | All sessions in block |
| 4.30 | 4.f | Action B - Block Both: Accuracy predicting action identity vs touch: test vs shuffle | Paired t-test | 32 sessions (8 mice) | 28.37 | 3.15e-23 | * | All sessions in block |
| 4.31 | 4.f | Action B - Block A: Accuracy predicting action identity vs touch: test vs shuffle | Paired t-test | 40 sessions (8 mice) | 49.75 | 8.71e-35 | * | All sessions in block |
| 4.32 | 4.f | Action B - Block B: Accuracy predicting action identity vs touch: test vs shuffle | Paired t-test | 40 sessions (8 mice) | 41.81 | 5.21e-34 | * | All sessions in block |
| 4.33 | 4.f | Action A vs Touch - Accuracy predicting action identity vs touch: Block Both vs Block A | Independent t-test | 72 sessions (8 mice) | 2.04 | 4.58e-2 | * | All sessions in block |
| 4.34 | 4.f | Action B vs Touch - Accuracy predicting action identity vs touch: Block Both vs Block A | Independent t-test | 72 sessions (8 mice) | 5.58 | 4.79e-7 | * | All sessions in block |
| 4.35 | 4.f | Action A vs Touch - Accuracy predicting action identity vs touch: Block Both vs Block B | Independent t-test | 72 sessions (8 mice) | 3.56 | 6.81e-4 | * | All sessions in block |
| 4.36 | 4.f | Action B vs Touch - Accuracy predicting action identity vs touch: Block Both vs Block B | Independent t-test | 72 sessions (8 mice) | 3.98 | 1.70e-4 | * | All sessions in block |
| 4.37 | 4.g | D1: Action A - Block Both: Accuracy predicting action identity vs touch: test vs shuffle | Paired t-test | 32 sessions (8 mice) | 16.13 | 1.01e-14 | * | All sessions in block |
| 4.38 | 4.g | D1: Action A - Block A: Accuracy predicting action identity vs touch: test vs shuffle | Paired t-test | 40 sessions (8 mice) | 21.96 | 4.84e-20 | * | All sessions in block |
| 4.39 | 4.g | D1: Action A - Block B: Accuracy predicting action identity vs touch: test vs shuffle | Paired t-test | 40 sessions (8 mice) | 21.97 | 1.84e-20 | * | All sessions in block |
| 4.40 | 4.g | D1: Action B - Block Both: Accuracy predicting action identity vs touch: test vs shuffle | Paired t-test | 32 sessions (8 mice) | 13.22 | 8.73e-13 | * | All sessions in block |
| 4.41 | 4.g | D1: Action B - Block A: Accuracy predicting action identity vs touch: test vs shuffle | Paired t-test | 40 sessions (8 mice) | 24.79 | 1.54e-21 | * | All sessions in block |
| 4.42 | 4.g | D1: Action B - Block B: Accuracy predicting action identity vs touch: test vs shuffle | Paired t-test | 40 sessions (8 mice) | 21.73 | 2.54e-20 | * | All sessions in block |
| 4.43 | 4.g | D2: Action A - Block Both: Accuracy predicting action identity vs touch: test vs shuffle | Paired t-test | 32 sessions (8 mice) | 15.95 | 1.30e-14 | * | All sessions in block |
| 4.44 | 4.g | D2: Action A - Block A: Accuracy predicting action identity vs touch: test vs shuffle | Paired t-test | 40 sessions (8 mice) | 22.54 | 2.33e-20 | * | All sessions in block |
| 4.45 | 4.g | D2: Action A - Block B: Accuracy predicting action identity vs touch: test vs shuffle | Paired t-test | 40 sessions (8 mice) | 29.44 | 3.18e-24 | * | All sessions in block |
| 4.46 | 4.g | D2: Action B - Block Both: Accuracy predicting action identity vs touch: test vs shuffle | Paired t-test | 32 sessions (8 mice) | 14.05 | 2.29e-13 | * | All sessions in block |
| 4.47 | 4.g | D2: Action B - Block A: Accuracy predicting action identity vs touch: test vs shuffle | Paired t-test | 40 sessions (8 mice) | 28.35 | 3.24e-23 | * | All sessions in block |
| 4.48 | 4.g | D2: Action B - Block B: Accuracy predicting action identity vs touch: test vs shuffle | Paired t-test | 40 sessions (8 mice) | 25.05 | 3.92e-22 | * | All sessions in block |
| 4.49 | 4.h, NR1-KO | Modeled total action rate as dependent on session | Linear mixed effect model | 12 sessions (3 mice) | Slope: -0.44 | 3.85e-8 | * | Data grouped by animal; modeled as random effect  Negative slope indicates drop with sessions |
| 4.50 | 4.h, littermate controls | Modeled total action rate as dependent on session | Linear mixed effect model | 20 sessions (4 mice) | Slope: 0.74 | 4.35e-3 | * | Data grouped by animal; modeled as random effect |
| 4.51 | 4.i, littermate controls | Action rate at Both reinforced block: A vs B | Paired t-test | 20 sessions (4 mice) | -1.92 | 0.074 | n.s. | All sessions in block |
| 4.52 | 4.i, littermate controls | Action rate at A reinforced block: A vs B | Paired t-test | 25 sessions (4 mice) | 3.72 | 1.45e-3 | * | All sessions in block |
| 4.53 | 4.i, littermate controls | Action rate at B reinforced block: A vs B | Paired t-test | 25 sessions (4 mice) | -2.29 | 0.033 | * | All sessions in block |
| 4.53 | 4.i, NR1-KO | Action rate at Both reinforced block: A vs B | Paired t-test | 12 sessions (3 mice) | -1.00 | 0.34 | n.s. | All sessions in block |
| 4.54 | 4.i, NR1-KO | Action rate at A reinforced block: A vs B | Paired t-test | 15 sessions (3 mice) | -4.79 | 2.89e-4 | * | All sessions in block  More B than A |
| 4.55 | 4.i, NR1-KO | Action rate at B reinforced block: A vs B | Paired t-test | 15 sessions (3 mice) | -2.70 | 0.017 | * | All sessions in block  More B than A |
| 4.56 | 4.j, NR1-KO | NR1-KO - Block Both: Accuracy predicting action identity: test vs shuffle | Paired t-test | 12 sessions (3 mice) | 19.16 | 2.63e-7 | * | All sessions in block |
| 4.57 | 4.j, NR1-KO | NR1-KO - Block A: Accuracy predicting action identity: test vs shuffle | Paired t-test | 15 sessions (3 mice) | 11.24 | 9.72e-5 | * | All sessions in block |
| 4.58 | 4.j, NR1-KO | NR1-KO - Block B: Accuracy predicting action identity: test vs shuffle | Paired t-test | 15 sessions (3 mice) | 18.03 | 2.26e-8 | * | All sessions in block |
| 4.59 | 4.j, littermate controls | Controls - Block Both: Accuracy predicting action identity: test vs shuffle | Paired t-test | 20 sessions (4 mice) | 14.27 | 9.80e-10 | * | All sessions in block |
| 4.60 | 4.j, littermate controls | Controls - Block A: Accuracy predicting action identity: test vs shuffle | Paired t-test | 25 sessions (4 mice) | 25.36 | 1.54e-15 | * | All sessions in block |
| 4.61 | 4.j, littermate controls | Controls - Block B: Accuracy predicting action identity: test vs shuffle | Paired t-test | 25 sessions (4 mice) | 24.38 | 8.49e-16 | * | All sessions in block |
| 4.62 | 4.j | Accuracy predicting action identity: NR1-KO vs controls | Independent t-test | 112 sessions (4+3 animals) | 2.29 | 2.48e-2 | * | All sessions |
| 5.1 | 5.k | Push ensemble activity: push actions vs pull action | Paired t-test | 21 sessions (10 mice) | 5.58 | 1.86-5 | * | 0.1 s before peak in calibration |
| 5.2 | 5.k | Pull ensemble activity: push actions vs pull action | Paired t-test | 21 sessions (10 mice) | -4.98 | 7.16e-5 | * | 0.1 s before peak in calibration |
| 5.3 | 5.l | Neural activity: push ensemble vs pull ensemble | Paired t-test | 21 sessions (10 mice) | 5.35 | 3.06e-5 | * | 0.1 s after stimulation |
| 5.4 | 5.l | Neural activity: push ensemble vs non-targeted | Paired t-test | 21 sessions (10 mice) | 5.55 | 1-97e-5 | * | 0.1 s after stimulation |
| 5.5 | 5.l | Neural activity: pull ensemble vs non-targeted | Paired t-test | 21 sessions (10 mice) | 0.24 | 0.81 | n.s. | 0.1 s after stimulation |
| 5.6 | 5.m | Neural activity: pull ensemble vs push ensemble | Paired t-test | 21 sessions (10 mice) | 4.33 | 3.26e-4 | * | 0.1 s after stimulation |
| 5.7 | 5.m | Neural activity: pull ensemble vs non-targeted | Paired t-test | 21 sessions (10 mice) | 4.48 | 2.30e-4 | * | 0.1 s after stimulation |
| 5.8 | 5.m | Neural activity: push ensemble vs non-targeted | Paired t-test | 21 sessions (10 mice) | 1.50 | 0.15 | n.s. | 0.1 s after stimulation |
| 5.9 | 5.n | SVM projection: post stim push ensemble | Paired t-test | 21 sessions (10 mice) | 3.53 | 2.10e-3 | * | 0.1 s before stim. And 0.1 s after stim. |
| 5.10 | 5.n | SVM projection: post stim pull ensemble | Paired t-test | 21 sessions (10 mice) | 4.85 | 9.63e-5 | * | 0.1 s before stim. And 0.1 s after stim. |
| 5.11 | 5.n | SVM projection: stim push vs stim pull | Paired t-test | 21 sessions (10 mice) | 4.90 | 8.57e-5 | * | 0.1 s before stim. And 0.1 s after stim. |
| 6.1 | 6.e | Force perturbation: congruent stimulation vs no-stim. | t-test | 12 sessions (9 mice) | 2.54 | 9.14e-3 | * | 0.1 s after stimulation |
| 6.2 | 6.e | Force perturbation: non congruent stimulation vs no-stim. | t-test | 12 sessions (9 mice) | 0.38 | 0.35 | n.s. | 0.1 s after stimulation |
| 6.3 | 6.e | Force perturbation: congruent stimulation vs non congruent stim. | t-test | 12 sessions (9 mice) | 2.27 | 1.64e-2 | * | 0.1 s after stimulation |
| 6.4 | 6.e | D1 – force perturbation: congruent vs non-congruent: 0 to 0.01 s | One-sided paired t-test | 24 sessions | 5.75e-1 | 2.85e-1 | n.s. | Selected interval after stimulation |
| 6.5 | 6.e | D1 – force perturbation: congruent vs non-congruent: 0.01 to 0.02 s | One-sided paired t-test | 24 sessions | 6.80e-1 | 2.52e-1 | n.s. | Selected interval after stimulation |
| 6.6 | 6.e | D1 – force perturbation: congruent vs non-congruent: 0.02 to 0.03 s | One-sided paired t-test | 24 sessions | 1.10 | 1.40-1 | n.s. | Selected interval after stimulation |
| 6.7 | 6.e | D1 – force perturbation: congruent vs non-congruent: 0.03 to 0.04 s | One-sided paired t-test | 24 sessions | 1.64 | 5.77e-2 | n.s. | Selected interval after stimulation |
| 6.8 | 6.e | D1 – force perturbation: congruent vs non-congruent: 0.04 to 0.05 s | One-sided paired t-test | 24 sessions | 2.15 | 2.10e-2 | * | Selected interval after stimulation |
| 6.9 | 6.e | D1 – force perturbation: congruent vs non-congruent: 0.05 to 0.06 s | One-sided paired t-test | 24 sessions | 2.45 | 1.11e-2 | * | Selected interval after stimulation |
| 6.10 | 6.e | D1 – force perturbation: congruent vs non-congruent: 0.06 to 0.07 s | One-sided paired t-test | 24 sessions | 2.54 | 9.07e-3 | * | Selected interval after stimulation |
| 6.11 | 6.e | D1 – force perturbation: congruent vs non-congruent: 0.07 to 0.08 s | One-sided paired t-test | 24 sessions | 2.65 | 7.23e-3 | * | Selected interval after stimulation |
| 6.12 | 6.e | D1 – force perturbation: congruent vs non-congruent: 0.08 to 0.09 s | One-sided paired t-test | 24 sessions | 2.78 | 5.28e-3 | * | Selected interval after stimulation |
| 6.13 | 6.e | D1 – force perturbation: congruent vs non-congruent: 0.09 to 0.10 s | One-sided paired t-test | 24 sessions | 2.78 | 5.31e-3 | * | Selected interval after stimulation |
| 6.14 | 6.e | D1 – force perturbation: congruent vs non-congruent: 0.10 to 0.11 s | One-sided paired t-test | 24 sessions | 2.51 | 9.85e-3 | * | Selected interval after stimulation |
| 6.15 | 6.e | D1 – force perturbation: congruent vs no stimulation: 0.10 to 0.11 s | One-sided t-test | 24 sessions | 2.29 | 1.57e-2 | * | Selected interval after stimulation |
| 6.16 | 6.e | D1 – force perturbation: non-congruent vs no stimulation: 0.10 to 0.11 s | One-sided t-test | 24 sessions | -0.30 | 6.15e-1 | n.s. | Selected interval after stimulation |
| 6.17 | 6.f | Force perturbation: congruent stimulation vs no-stim. | t-test | 9 sessions (5 mice) | 2.21 | 2.04e-2 | * | 0.1 s after stimulation |
| 6.18 | 6.f | Force perturbation: non congruent stimulation vs no-stim. | t-test | 19 sessions (5 mice) | 1.57 | 0.07 | n.s. | 0.1 s after stimulation |
| 6.19 | 6.f | Force perturbation: congruent stimulation vs non congruent stim. | t-test | 19 sessions (5 mice) | 0.09 | 0.19 | n.s. | 0.1 s after stimulation |
| 6.20 | 6.f | D2 – force perturbation: congruent vs non-congruent: 0 to 0.01 s | One-sided paired t-test | 18 sessions | 7.22e-1 | 2.40e-1 | n.s. | Selected interval after stimulation |
| 6.21 | 6.f | D2 – force perturbation: congruent vs non-congruent: 0.01 to 0.02 s | One-sided paired t-test | 18 sessions | 2.27.-1 | 4.11e-1 | n.s. | Selected interval after stimulation |
| 6.22 | 6.f | D2 – force perturbation: congruent vs non-congruent: 0.02 to 0.03 s | One-sided paired t-test | 18 sessions | 1.76e-1 | 4.31e-1 | n.s. | Selected interval after stimulation |
| 6.23 | 6.f | D2 – force perturbation: congruent vs non-congruent: 0.03 to 0.04 s | One-sided paired t-test | 18 sessions | 3.68e-1 | 3.59e-1 | n.s. | Selected interval after stimulation |
| 6.24 | 6.f | D2 – force perturbation: congruent vs non-congruent: 0.04 to 0.05 s | One-sided paired t-test | 18 sessions | 6.45e-1 | 2.65e-1 | n.s. | Selected interval after stimulation |
| 6.25 | 6.f | D2 – force perturbation: congruent vs non-congruent: 0.05 to 0.06 s | One-sided paired t-test | 18 sessions | 7.96e-1 | 2.19e-1 | n.s. | Selected interval after stimulation |
| 6.26 | 6.f | D2 – force perturbation: congruent vs non-congruent: 0.06 to 0.07 s | One-sided paired t-test | 18 sessions | 8.41e-1 | 2.06e-1 | n.s. | Selected interval after stimulation |
| 6.27 | 6.f | D2 – force perturbation: congruent vs non-congruent: 0.07 to 0.08 s | One-sided paired t-test | 18 sessions | 9.48e-1 | 1.78e-1 | n.s. | Selected interval after stimulation |
| 6.28 | 6.f | D2 – force perturbation: congruent vs non-congruent: 0.08 to 0.09 s | One-sided paired t-test | 18 sessions | 1.18 | 1.28e-1 | n.s. | Selected interval after stimulation |
| 6.29 | 6.f | D2 – force perturbation: congruent vs non-congruent: 0.09 to 0.10 s | One-sided paired t-test | 18 sessions | 1.49 | 7.78e-2 | n.s. | Selected interval after stimulation |
| 6.30 | 6.f | D2 – force perturbation: congruent vs non-congruent: 0.10 to 0.11 s | One-sided paired t-test | 18 sessions | 1.78 | 4.70e-2 | * | Selected interval after stimulation |
| 6.31 | 6.f | D2 – force perturbation: congruent vs no stimulation: 0.10 to 0.11 s | One-sided t-test | 18 sessions | 1.16 | 3.03e-2 | * | Selected interval after stimulation |
| 6.32 | 6.f | D2 – force perturbation: non-congruent vs no stimulation: 0.10 to 0.11 s | One-sided t-test | 18 sessions | 0.33 | 3.74e-1 | n.s. | Selected interval after stimulation |

Extended Data Table 2 – Summary of statistical tests in Extended Data Figures

| Table ref. | Figure Panel | Group | Statistical test | Sample size | Test Statistic | *P* value | Sig. | Notes |
| --- | --- | --- | --- | --- | --- | --- | --- | --- |
| 2.1 | 2.f | Push weighted: D1 vs D2 | Paired t-test | 63 sessions (8 mice) | -0.57 | 0.57 | n.s. | -0.5 s to peak and at peak (0 s) |
| 2.2 | 2.f | Push weighted: D1 vs all MSNs | Paired t-test | 63 sessions (8 mice) | 0.49 | 0.62 | n.s. | -0.5 s to peak and at peak (0 s) |
| 2.3 | 2.f | Push weighted: D2vs all MSNs | Paired t-test | 63 sessions (8 mice) | 1.38 | 0.17 | n.s. | -0.5 s to peak and at peak (0 s) |
| 2.4 | 2.f | Pull weighted: D1 vs D2 | Paired t-test | 73 sessions (8 mice) | -0.36 | 0.72 | n.s. | -0.5 s to peak and at peak (0 s) |
| 2.5 | 2.f | Pull weighted: D1 vs all MSNs | Paired t-test | 73 sessions (8 mice) | -1.15 | 0.25 | n.s. | -0.5 s to peak and at peak (0 s) |
| 2.6 | 2.f | Pull weighted: D2vs all MSNs | Paired t-test | 73 sessions (8 mice) | -0.83 | 0.41 | n.s. | -0.5 s to peak and at peak (0 s) |
| 4.1 | 4.g, littermate controls | Action rate at Both reinforced block: push vs pull | Paired t-test | 20 sessions (4 mice) | -0.05 | 0.96 | n.s. | All sessions in block |
| 4.2 | 4.g, littermate controls | Action rate at Pull reinforced block: pull vs push | Paired t-test | 25 sessions (4 mice) | -3.33 | 3.56e-3 | * | All sessions in block |
| 4.3 | 4.g, littermate controls | Action rate at Push reinforced block: push vs pull | Paired t-test | 25 sessions (4 mice) | 2.60 | 1.7e-2 | * | All sessions in block |
| 4.4 | 4.g, NR1-KO | Action rate at Both reinforced block: push vs pull | Paired t-test | 12 sessions (3 mice) | -1.0 | 0.34 | n.s. | All sessions in block |
| 4.5 | 4.g, NR1-KO | Action rate at Pull reinforced block: pull vs push | Paired t-test | 15 sessions (3 mice) | -2.70 | 1.7e-2 | * | All sessions in block |
| 4.6 | 4.g, NR1-KO | Action rate at Push reinforced block: push vs pull | Paired t-test | 15 sessions (3 mice) | -4.79 | 2.89e-4 | * | All sessions in block |
