# Supplementary material for: Striatal ensembles specify and control granular forelimb actions
Source: bioRxiv. 2025 Dec 8:2025.12.03.692128. Preprint. [Version 1] doi: 10.64898/2025.12.03.692128 (PMC12713657; doi:10.64898/2025.12.03.692128)
Supplement: Supplement 3 [file media-3.docx]

**Supplementary Information**

**Two-action isometric task**

**Setup**

The mouse was head-fixed and held in a 3D printed plastic cup. A spout to deliver sucrose reinforcement was placed in tongue reaching distance. The mouse used the right forelimb to interact with an immobile joystick – 3mm screw -mounted on 3-D printed plastic frames that were attached to load cell sensors (Phidgets single point load cell 3139_0). There were two load sensors attached to the joystick: one measured push/pull forces, and the other measured left/right forces. The mouse rested the left forelimb on an auxiliary pole, where two load sensors were attached: one sensor measured push/pull forces and the other measured vertical force. The cup holding the mouse was mounted on one additional load sensor that measured the animal’s body movement. Data from the load sensors was recorded at 1kHz and processed with scientific board (“Scientific Board v1.3”) with a quad load cells module (“Quad load cell v1.2”) from Champalimaud Foundation Scientific (CFS) Hardware Platform. (Note: we used updated boards in the “Adaptive Isometric task”.) This board also computed when the force measured on the sensors crossed experimenter-defined thresholds. The CFS Hardware Platform’s “Load cells visualizer” software was used to visualize and save the data. We used pyControl boards^3^ (the “Lickometer v1.0”) to detect licking and touching of the joystick and auxiliary pole. A camera (Teledyne FLIR Flea3 1.3 MP, Mono: FL3-U3-13Y3M-C) collected video at 30 or 60 fps and were controlled with Bonsai software^4^.

The task and reinforcement were implemented as follows. Another pyControl board (the “Breakout 1.2” board) controlled the task, recorded task events at 1kHz, and delivered sucrose as reinforcement. For reinforcement, the pyControl board opened a solenoid for ~5μl to deliver 10% sucrose solution to the animal. Explicitly, when the solenoid opened, sucrose flowed with gravity through tubing and finally through a blunt needle (16G) - spout - mounted proximal to the animal’s mouth. Every session before behavioral training, we calibrated the time the solenoid needed to open in order to deliver 5μl of sucrose. The 10% sucrose solution was made fresh every week using the water provided to mice by the Institute for Comparative Medicine (ICM) facility. Another pyControl board (the “Lickometer v1.0”) detected licking of the sucrose delivery needle. The hardware for the setup is shown in Extended Data Fig. 7a.

**Training**

Mice were habituated to head-fixation with at least 15 minutes on a running wheel for 4 days and food restricted to a maximum of 85-80% of their initial weight. Then training of the two-action isometric task started as described in protocols.io (“Behavior training”). The task required the mice to push or pull an immobile, pressure-sensitive joystick in a self-paced manner. The task defined a task action as when the exerted force in either the push or pull direction was greater than the task threshold for a larger time interval than the task duration. With training, the task threshold and duration were progressively modified (see details in Extended Data Table 2). The task identified the animal’s exerted force as an action when the force rose above the task threshold, in either the push or pull direction. The task defined the start and end of the animal’s action as the times when the exerted force rose above and fell below the task threshold. Reinforcement for a task action was delivered with a delay from the end of the action. The reinforcement delay was also progressively modified with training (see details in Extended Data Table 2). The task also used a 3g threshold to define when mice were quiescent enough for a trial to initiate so that reinforcement could be triggered based on task actions (see details in Extended Data Table 2).

The structure of trials was as follows:

1) First, there was an inter-trial interval (ITI) that lasted 1 second during which no reinforcement was delivered. At the end of ITI, if the force was below the 3g threshold, the trial initiated. If the force was above the 3g threshold, another ITI was initiated.

2) Next, a trial initiated. During the trial, the execution of a task action (either a push or a pull that crossed the task threshold for longer than the task duration) triggered delayed reinforcement. The trial concluded at the moment of reinforcement delivery. If the animal initiated an action but its duration was shorter than the task duration, the animal “failed” the trial, and the trial immediately concluded when the force dropped below the task threshold. After the trial concluded, the task re-entered the ITI (back to step 1 above).

This task was trained in three blocks, each of which used a different reinforcement schedule:

1) In the first block - “Block Both” - both actions were reinforced. At the end of Block Both, “action A” was defined as the action (push or pull) that the animal produced least frequently, and “action B” was defined as the action (push or pull) that the animal produced most frequently.

2) In the second block – “Block A” - only action A was reinforced.

3) In the third block – “Block B” - only action B was reinforced.

In the reinforcement schedule for Block Both, both push and pull actions were reinforced. Each session concluded after animals executed 50 task pushes and 50 task pulls, or after 30 minutes elapsed.

The task threshold, duration and reinforcement delays were modified progressively (see Extended Data Table 2). Animals began training with task parameters called “Parameters label: 1” below. The session’s task parameters advanced to the next set of parameters (Parameters label: 2) on the session after the animal performed more than 50 task actions in a session. Block Both concluded when the animal performed more than 50 task actions in each of 2 consecutive sessions in “Parameters label: 3”.

In Block A and B, only one task action was reinforced (action A for block A, action B for block B). Each session concluded when animals executed 200 reinforced task actions or after 30 minutes elapsed. In these blocks, the task threshold, duration and reinforcement delays changed progressively (see Extended Data Table 2). Animals began training with task parameters called “Parameters label: 1”. The session’s task parameters advanced to the next set of parameters (Parameters label: 2) on the session after the animal performed more than 50 task actions in a session. Each block concluded when the action proportion for the reinforced task action was 80% or higher when averaged over 3 consecutive sessions in “Parameters label: 3”. Action proportion for the reinforced task action was calculated as the number of reinforced task actions divided by the total number of executed task actions for action A and B together. Block B started on the session after the final session of Block A. Block B’s conclusion marked the end of training, and animals were fed ad libidum.

For RGS9-LCre::Grin1tm1Yql homozygous (referred to as Striatal NR1-KO) and Cre negative littermates were trained as described in protocols.io (“Behavior training”). The maximum number of sessions per block was defined as 1.2 times longer than the third animal to reach reinforcement change criteria. “Give up” criteria was defined as performing less than 10 3g cross actions that crossed 3g for ~50 ms (sum of push and pull) on average over 3 consecutive sessions. If animals reached the maximum number of sessions or the give up criteria, mice were moved to next phase of training.

**2-photon microscope**

The optical setup was composed of two femtosecond pulse lasers and a custom-modified two-photon laser scanning microscope (Ultima In Vivo, with Neuralight3D, Bruker Corporation, Billerica, Massachusetts) equipped with a 8 kHz resonant scanner. The laser source for imaging was a pulsed Ti:sapphire laser (Chameleon Vision-S, Coherent, Inc., Saxonburg, Pennsylvania). Its wavelength was tuned to 920 nm for GCaMP6s or GCaMP6f imaging. The laser power was controlled with a Pockel cell (350–105-BK Pockels cell, 302RM controller, Conoptics, Inc., Danbury, Connecticut). The laser beam was expanded by a 1:2 telescope (Thorlabs GBE02-B). The beam was further scaled in the microscope by a 1:1.33 telescope before it was coupled into a scan lens (f = 75 mm), a tube lens (f = 180 mm) and the objective lens (16X 0.8NA N16XLWD-PF Nikon Corporation, Tokyo, Japan). The laser could also be directed to a non-resonant scanning path where both X and Y scanning were controlled by galvanometric mirrors. The fluorescence signal from the sample was collected through the objective lens, split from the IR with a 670 nm long-pass dichroic (ZT670rdc, Chroma Technology Corp., Bellows Falls, Vermont) and coupled into a liquid light guide assembly that transmits the light to a detection unit. This light was then split by a 2” dichroic mirror (HQ565dcxr, 565 nm long pass, Chroma Technology Corp., Bellows Falls, Vermont), with the “green” and “red” light each directed to GAsP PMTs, through 2” bandpass filters 510/20–2P, and 607/45–2P respectively (Chroma Technology Corp., Bellows Falls, Vermont).

The optical path for the photostimulation was largely independent from the imaging path and it was only used for photostimulation experiments. The laser source for photostimulation was a low repetition rate (1 MHz) amplified laser (Monaco 1035–40, Chameleon Vision-S, Coherent, Inc., Saxonburg, Pennsylvania), operating at 1035 nm wavelength. Its power was controlled by an integrated acousto-optic modulator. The beam was expanded by a telescope (1:2 telescope (Thorlabs GBE02-B), and directed into the microscope. The photostimulation laser could be steered either simply by a dedicated pair of galvanometers, or coupled to a customized light path that included a spatial light modulator (Bruker Neuralight 3D, Billerica, Massachusetts). The telescope relay system within the Neuralight 3D was modified with custom lenses to provide additional corrections for lateral color, but is otherwise functionally identical to the original module. Finally, this beam was combined and made coaxial with the imaging beam just before the scan lens of the microscope with a 1030 nm shortpass dichroic (T1030SP, 1030 nm short pass, Chroma Technology Corp., Bellows Falls, Vermont).

The imaging and photostimulation were controlled by a combination of PrairieView (Bruker Corporation, Billerica, Massachusetts) and custom software running under MATLAB (The Mathworks, Inc. Natick, Massachusetts) and Python. Custom software was developed to control what photostimulation pattern the microscope should deliver and when to trigger stimulation in closed-loop with the animal’s ongoing behavior. It interfaced with PrairieView using the PrairieLink API.

The typical imaging power was < 50 mW, and could be up to 80 mW for deeper than ~ 250 μm below the terminus of the GRIN lens. Images were acquired using Prairie View software (Bruker Corporation, Billerica, Massachusetts) at 30 Hz. The functional data was extracted from a square of 512 x 512 pixels of the following size: for Fig. 1-4 we used 1.5x zoom over 496.1 μm x 496.1 μm; in Fig. 5-6 we used 1.5x zoom over 550.4 μm x 550.4 μm and 2x zoom over 412.7 μm x 412.7 μm.

Most mice were imaged for the entire duration of training, with some having the same FOV tracked over several days. ROIs identified in previous days were used to find the same FOV and custom-made code was written to track same ROIs across different days. Data shown in figures 1,2,3 are from mice that were trained while simultaneously imaged. Functional recordings were taken for the entire duration of behavior training whilst structural images and z-stacks were taken at the end of session with “green” and “red” light each directed to respective GAsP PMTs. These images were then used to identify identity of ROIs (Extended Data Fig.1).

A custom-made computerized goniometer was used to subtly and reproducibly angle the head of the mouse such that the imaging lens was orthogonal to the beam path.

**Adaptive Isometric task – Setup**

We developed a closed-loop system that triggered stimulation based on the force that the animal exerted on the immobile joystick. The following features were required in this system:

1) Select, on single-trial basis, threshold crossing triggering stimulation

2) Trigger stimulation with millisecond latency

3) Specify, on single-trial basis, pattern of 2-photon stimulation

Features 1) and 2) required an improved hardware system for processing force sensors, while 3) required Bruker software engineers to update the 2-photon microscope’s flexibility for specifying patterns of 2-photon stimulation.

The hardware system to accomplish 1) and 2) used hardware boards (the HARP load cells acquisition v1.1 board combined with the HARP load cell interface v1.1 board, both from the Champalimaud Foundation Scientific (CFS) Hardware Platform https://www.cf-hw.org/harp/load-cells) that could evaluate in parallel when the force exerted on the joystick crossed 6 different thresholds specified by the experimenter at the start of the experimental session were used for this setup. In the stimulation experiments, 6 thresholds were defined: 3g, 4g, and 6g thresholds for push and pull actions. To select which force threshold would trigger stimulation on a particular trial, and we gated the trigger signal to be deployed within trials, while minimizing the delay between threshold crossing and the onset of 2-photon stimulation. We built a simple hardware board consisting of a MUX and an AND gate to select and allow the trigger reach the microscope. The MUX and the AND gate were controlled by custom Matlab scripts – run on computer that controlled the microscope. The Matlab scripts determined which force threshold should serve as the trigger signal on each trial and when to enable trigger. This required Matlab to receive the ITI signal from the task and the output signal from the. This digital communication from the task boards to the computer and from the computer to the MUX and AND gate was implemented with a simple NIDAQ DIO board (USB-6001).

Feature 3) was accomplished with a newly implemented software function in the PrairieLink API of the Bruker microscope. This function allowed to specify 2-photon stimulation patterns in a dynamic manner in the middle of an imaging experiment. Schematics of the setup presented in Fig.5.

**Definition of actions**

We use the following actions for analysis:

- Task action: push and pull actions that were identified by the task hardware to trigger reinforcement and that were changed progressively across training (details in Extended Data Table 2). Task actions were identified from force traces sampled at 1kHz. The action interval was defined as between the rise and fall crossings of a force threshold.
  - Task actions were analyzed in Fig. 3bc, Extended Data Fig. 4a-f.
- 3g cross action: push and pull that cross the 3g threshold for at least ~66ms (2 imaging frames), with the touch sensor registering joystick touch for at least 100ms (3 imaging frames) before the 3g cross. The touch requirement ensured the actions are isometric. The action interval was defined as between the rise and fall crossings of 3g. Trials were identified from smoothed force traces that were matched to the 30Hz sampling rate of 2p imaging. To downsample the 1kHz raw force trace to 30Hz, we averaged 33ms bins of force centered at the timestamp of each imaging frame.
  - 3g cross actions were analyzed in Fig. 2, 3, 4d-f, i, 5, and 6, as well as Extended Data Fig. 2, 3, 5, and 6.
- 6g cross action: 3g cross actions that have a force peak above 6g. 6g was the final force threshold in each block of training the two-action isometric task.
  - 6g cross actions were analyzed in Fig. 1c-j and 4h,i, as well as Extended Data Fig. 4g-i.
- Touch: Joystick touch with force lower than 2g for more than 1s.
  - Touch actions were analyzed in Fig. 2h,i and Extended Data Fig. 2.
- Lick: Bout of licks with inter-lick interval less than 0.5s, with at least 5 licks, and without overlap with 3g cross actions.
  - Lick actions were analyzed in Fig. 2h,i and Extended Data Fig. 2.

We also analyzed selected trials of push and pull actions that met additional criteria:

- Pre-isolated 3g cross actions: 3g cross actions that have no other 3g cross actions in the 0.5 seconds before 3g cross.
  - These actions were analyzed in Fig. 2c-g, j-n and 3, as well as Extended Data Fig. 2 and 3.
- Post-isolated 3g cross actions: 3g cross actions that have no other 3g cross actions in the 0.5 seconds after 3g cross.
  - These actions were analyzed in Fig. 2e-g.
- Matched actions: A matching procedure (see “Matching trials across actions” below) selected trials to match features across push and pull.
  - These actions were analyzed in Fig. 1f-j, Extended Data Fig. 5,6.

**Matching trials across actions**

We sought to test if neural activity encoded push and pull actions that had matched trial-averaged features (Fig. 1f-j, Extended Data Fig. 5,6). We defined tolerances for features to be matched across actions. In Fig. 1f-j, average force was within 0.1g across actions. In Extended Data Fig. 5,6, tolerances were the following:

- Peak force was within 0.1g
- The two-axis force vector magnitude at the time of peak push/pull force was within 0.2g
- Action duration was within 30ms
- Lick rate during actions was within 0.5 Hz
- Lick rate at peak push/pull force was within 0.5 Hz
- Lick probability during actions was within 0.1
- Lick probability at peak push/pull force was within 0.1

Then we applied a matching procedure that iteratively removed trials from selection to achieve matching within tolerance, trying to remove as few trials as possible. We first z-scored all features (e.g. peak force, action duration, etc) across all trials (pooling across actions). Then we executed our matching algorithm:

1. Define action 1 as the action with fewer trials remaining in selection, and action 2 as the other. (We will remove trials from action 2.)
2. Calculate a target vector as the trial-averaged feature vector of action 1 minus the trial-averaged feature vector of action 2. (We want to remove trials so action 2’s features move in the direction of the target vector.)
3. Zero out features of the target vector that are within-tolerance. (We don’t want features that are within-tolerance to influence trial removal.)
4. Calculate the dot product of action 2 trials’ feature vectors with the target vector.
5. Remove the trial with the smallest dot product.
6. If not within matching tolerance for all features, repeat steps with the remaining trials.

In Extended Data Fig. 5,6, we pre-selected trials before the matching procedure such that the time between solenoid opening and force threshold crossing was at least 4 seconds, ensuring that identified actions were not part of sucrose consumption.

**Time course of action force**

We visualized the average time course of joystick force locked to action events (also known as a peri-event time histogram, or PETH).

- For an example session in Fig. 1c, we calculated the trial-averaged force for 6g cross push and pull actions locked to 6g cross.
- For pre-isolated 3g cross actions, we calculated trial-averaged force locked to force peak (Fig. 2c) and 3g cross (Fig. 3a, Extended Data Fig. 3).
- In Fig. 2f and Extended Data Fig. 3c, the force from -4 seconds to 0 seconds is from pre-isolated 3g cross actions, and the force from 0 seconds to 4 seconds is from post-isolated 3g cross actions. This visualizes trials used to predict action identity from neural activity.

**Quantification of actions across reinforcement**

We present multiple quantifications of action performance across our reinforcement schedule:

- 6g cross actions
  - We quantified the action rate (actions per minute) pooling the selected sessions within each reinforcement block (Fig. 1d, Fig. 4i, Extended Data 4g). In Fig. 1d and Extended Data Fig. 4g., we pooled data by whether push or pull was reinforced, irrespective of their label as A or B.
  - We quantified the overall action rate across selected sessions (Fig. 4h).
- Task actions
  - We quantified the action rate (Fig. 4b, Extended Data Fig. 4h) and the proportion of task actions with each identity across selected sessions (Fig. 4c, Extended Data Fig. 4i).
  - We showed the action rate across all sessions and animals (Extended Data Fig. 4a-f).
- 3g cross actions
  - We quantified the following metrics across selected sessions for 3g cross actions (Extended Data Fig. 5a-h) and matched 3g cross actions (Extended Data Fig. 5k-r):
    - number of actions per session
    - magnitude of the two-dimensional force vector at time of peak push/pull force
    - average force over the action interval (between rise and fall crossing of 3g)
    - peak push/pull force
    - average lick probability over the action interval
    - average duration of the action interval
    - action rate
    - action proportion

**EMG analysis**

We analyzed filtered 1kHz EMG of four forelimb muscles (see “EMG recordings”) for 6g cross actions with force matched within 0.1g across actions. We z-scored each EMG channel using the entire session’s recording, and time-locked analysis to the 6g cross event.

To analyze the dimension of muscle activity that captures the most variance across time and actions, we performed principal components analysis (PCA) using the Python package “sklearn.decomposition.PCA.” For each session, we concatenated a 10 second window of trial-averaged force centered at 6g cross for each action. Thus, we applied PCA on a 2D matrix of size (num_muscles) x (num_actions*num_samples), with num_muscles=4, num_actions=2, and num_samples=10000. We analyzed the projection of EMG onto the first principal component (PC1, Fig. 1h) for each action.

We quantified the similarity of EMG across actions by comparing average and differential EMG modulation. We used the trial-averaged time course of force for each action in a 0.4 second window centered at 6g cross, and we defined the average across actions as “average modulation,” and the averaged difference across actions (push minus pull divided by 2) as “differential modulation.” We compared the variance of average and differential modulation in the 0.4 second window (Fig. 1i).

We tested whether EMG exhibited specific patterns for each action. We compared the vector magnitude of EMG difference across actions to the difference within each individual action. Concretely, for each session, we split trials for each action into two halves and calculated the force PETH for each half. At each time point locked to 6g cross, we calculated the vector magnitude of difference across halves within an action, as well as the difference across actions using the PETH from one half. We compared across-action and within-action difference in the 100ms centered at 6g cross (Fig. 1j).

**Distinguishing D1- and D2-MSNs**

We developed an automated thresholding method to identify D1- and D2-MSNs based on fluorophore expression (Extended Data Fig. 1), combined with transgenic labeling and functional/structural 2-photon imaging. Functional imaging (green channel) was used to detect ROIs with Suite2p, while structural imaging (red channel) determined co-localization of cell-type–specific fluorophores. The overall workflow is outlined in Extended Data Fig. 1a. The method involved three main steps: (1) aligning and processing structural and functional data, (2) labeling ROIs manually (ground truth) and automatically using red-channel thresholds, and (3) comparing manual and automatic labeling.

**1) Structural data processing**
Functional data were motion-corrected and ROIs extracted using Suite2p (Extended Data Fig. 1b). As fluorophore expression is soma-restricted, only pixels within 12-pixel diameter of each ROI center were used. Structural images (red channel, 1000 frames) were motion-corrected against the functional template, then background-subtracted using a 25-pixel rolling ball filter in ImageJ (Extended Data Fig. 1c,d).

**2) ROI labeling**
ROIs near the edge of the GRIN lens were excluded to avoid aberrations; the lens center was estimated from the red channel by summing projections and taking median coordinates above the 20th percentile. Valid ROIs fell within a circle around this center.

a. Manual labeling:
Manual labels were assigned on average, motion-corrected, background-subtracted structural images overlaid with functional ROIs (Extended Data Fig. 1e). Labels were “red,” “non-red,” or “no-ID” if uncertain. Each mouse had at least two sessions labeled three times; only ROIs consistently labeled across all folds were retained (Extended Data Fig. 1f,g). Thresholds of 0.7 (red) and 0.3 (non-red) were determined for automatic labeling.

b. Automatic labeling:
Structural images were processed with Ilastik^1^ (v1.3.3) to estimate per-pixel probability of being red (Extended Data Fig. 1h). ROIs with >0.7 red pixels were labeled “red,” <0.3 as “non-red,” and 0.3–0.7 as “no-ID” (Extended Data Fig. 1i).

**3) Manual vs. automatic comparison**
Label accuracy, swapping errors, and single-method errors were quantified across threshold pairs to optimize performance (Extended Data Fig. 1j–m, 9k). Thresholds of 0.3/0.7 (non-red/red) were used for general characterization (Figs. 2,3), and 0.45/0.55 thresholds were used for holographic stimulation experiments (Figs. 4,5).

**Time course of neuronal activity**

We visualized and analyzed the time course of neuronal activity locked to action events.

- In Fig. 2b, we showed an example trace of neuronal activity averaged over all MSNs, D1-MSNs (n=21), and D2-MSNs (n=21). For the visualization, we chose a random subset of 21 D1-MSNs so the number was matched to the 21 identified D2-MSNs.
- In Fig. 2c, we analyzed the PETH averaged over all identified MSNs, D1-, and D2-MSNs locked to force peak of pre-isolated 3g cross actions.
- In Fig. 2d, we visualized the PETH of example D1- and D2-MSNs locked to force peak of pre-isolated 3g cross actions.

**Support Vector Machine (SVM) decoder model**

To test whether MSNs encoded specific actions, we built a SVM classifier that used neural activity to predict action identity on single trials. The SVM classifier with linear kernel was built using the ‘scikit-learn’ package in Python (sklearn.svm.linearSVC). Concretely, we constructed a population activity vector for each trial, where each entry was the activity of one neuron. A neuron’s activity was the average ΔF/F_0_ in 5 imaging frames (~167ms) centered at the time of action events. We used this population activity vector to predict the action identity between a pair of actions. For touch and lick actions, we defined the action event as the time in the middle of the action interval (Fig. 2h,i and Extended Data Fig. 5i,s and 6c,g).

We trained and tested the model as follows. To avoid biasing the SVM to predict the action with more trials, we weighted trials by 1/number of trials. There needed to be at least 20 trials of each action per session in order to run the SVM analysis. We split the trials so that a random 90% of trials were “train trials” to fit the model and the remaining 10% of trials were “test trials” to test the model’s performance. We performed this random train-test split 100 times. For each fold, we used the train data to fit the model (including optimizing the regularization parameter), and we evaluated the model’s accuracy on the test data.

Within each fold, we optimized the regularization parameter so that models would not overfit to training data. We split the training data into ‘sub-training data’ and ‘sub-testing data’ in 10 sub-folds to optimize the regularization parameter. 80% of training data was randomly selected for sub-training data, and the remaining 20% of data was sub-testing data. We swept the regularization parameter over a range of values (the ‘C’ parameter was swept between 1e-5 to 100), and for each regularization value, we fit a model on the sub-training data and tested it on the sub-testing data. We selected the regularization parameter that resulted in the best model performance on the sub-test data, averaged across the 10 sub-folds. Note that our procedure could select a different regularization value for different folds of the train-test split of data.

We evaluated the overall accuracy as the fraction of trials for which the action label was correctly predicted. In addition, we evaluated the model’s accuracy for each action separately, e.g. we evaluated the percent of trials that were correctly predicted for push action separately and for pull action separately (Extended Data Fig. 5j, t and 6d, e, i, j). We compared the model’s performance to a shuffle model’s performance. The shuffle model was trained on data where the action label was shuffled but was tested on data where the action label was not shuffled.

**SVM decoding across sessions**

We reported the accuracy of decoding action identity for individual sessions across reinforcement and pooling sessions.

- SVM accuracy across each pair of actions, pooling the selected sessions across all reinforcement blocks:
  - Fig. 2h decoded all MSNs. For push and pull, 3g cross actions were used, and neural activity was centered at force peak.
    - Extended Data Fig. 5i is the same as Fig. 2h, but separating by action A and B rather than push and pull.
    - Extended Data Fig. 5s is the same as Extended Data. Fig 5i but for matched 3g cross actions.
  - Fig. 2i is the same as Fig. 2h, but decoding D1- and D2-MSN activity.
    - Extended Data Fig. 6c is the same as Fig. 2i, but separating by action A and B rather than push and pull.
    - Extended Data 6g is the same as Extended Data Fig. 6c but for matched 3g cross actions.
- SVM accuracy for each selected session across reinforcement:
  - Fig. 4d decoded all MSNs at force peak for 3g cross actions.
    - Extended Data Fig. 5u is the same as Fig. 4d, but for matched 3g cross actions.
    - Extended Data Fig. 5j left is the same as Fig. 4d, but reporting accuracy separately for action A trials and action B trials.
    - Extended Data Fig. 5t left is the same as Extended Data Fig. 5j, except for matched 3g cross actions.
  - Fig. 4e left is the same as 4d, but decoding D1- and D2-MSN activity.
    - Extended Data Fig. 6h left is the same as Fig. 4e left, but for matched 3g cross actions.
    - Extended Data Fig. 6e is the same as Fig. 4e left, but reporting accuracy separately for action A trials and B trials.
    - Extended Data Fig. 6j is the same as Extended Data Fig. 6e, but for matched 3g cross actions.
  - Fig. 4f left decoded all MSNs at force peak for 3g cross actions. One SVM predicted action A versus touch, and another SVM predicted action B versus touch.
    - Extended Data Fig. 5v left is the same as Fig. 4f left, but for matched 3g cross actions.
    - Extended Data Fig. 6f is the same as Fig. 4f, but for D1- and D2-MSNs.
    - Extended Data Fig. 6k is the same as Fig. 6f, but for matched 3g cross actions.
- SVM accuracy pooling selected sessions within each reinforcement block:
  - Fig 4e right decoded D1- and D2-MSNs at force peak for 3g cross actions.
    - Extended Data Fig. 6h right is the same as Fig. 4e right, but for matched 3g cross actions.
  - Fig. 4j is the same as Fig. 4e right, but decoding all striatal for NR1-KO and littermate control mice.
  - Fig. 4f right decoded MSNs at force peak for 3g cross actions. One SVM predicted action A versus touch, and another SVM predicted action B versus touch.
    - Extended Data Fig. 5v right is the same as Fig. 4f, but for matched 3g cross actions.
    - Extended Data 6l is the same as Fig. 4g, but for matched 3g cross actions.
  - Fig. 4g is the same as Fig. 4f right, but for D1- and D2-MSNs.
  - Extended Data Fig. 5j right decoded all MSNs for 3g cross actions, reporting accuracy separately for action A trials and action B trials.
    - Extended Data Fig. 5t right is the same as Extended Data Fig. 5j right, but for matched 3g cross actions.
    - Extended Data Fig. 6d is the same as Extended Data Fig. 5j right, but decoding D1- and D2-MSNs.
    - Extended Data Fig. 6i is the same as Extended Data Fig. 6d, but for matched 3g cross actions.

**Time course of action decoding**

We used the SVM to analyze the time course of action decoding. We extracted neural activity centered at specific time points relative to action events.

- In Fig. 2e-g, we analyzed non-overlapping 5 frame bins locked to force peak. The bin at time 0 was centered at force peak. From -4 seconds to 0 seconds, the SVM was applied on trials from pre-isolated 3g cross actions. From after 0 seconds to 4 seconds, the SVM was applied on trials from post-isolated 3g cross actions. The SVM used all MSNs in Fig. 2ef and matched numbers of D1- or D2-MSNs in Fig. 2g.
- In. Fig. 3a, we analyzed 5 frame bins with a temporal step of 2 frames (~67ms) locked to 3g cross of pre-isolated 3g cross actions. This enabled decoding neural activity during a time window with no action occurring.
- In Extended Data Fig. 3b, we compared the time course of action decoding locked to force peak versus locked to 3g cross.
- In Fig. 3c, we compared accuracy of the decoder in Fig. 3a (which we termed the “instantaneous decoder” because a new decoder is optimized for each instant in time) to a “3g cross decoder” that was only optimized on neural activity at time 0 seconds (i.e. the 5 frame bin centered at 3g cross). Concretely, on every decoder training fold using neural activity centered at time 0 seconds, we applied the SVM on neural activity of test trials at all time points sweeping from -0.5 seconds to 0 seconds.
- In Fig. 3d, we normalized the data in Fig. 3c as follows. We subtracted the accuracy of the 3g cross decoder from the instantaneous decoder (shown in Extended Data Fig. 3e). We then divided this delta accuracy by the range of the 3g cross decoder’s session-averaged accuracy in the time window from -0.5 seconds to 0 seconds. Concretely, this range was the maximum value minus the minimum value of the session-averaged 3g cross decoder accuracy shown in Fig. 3c.
- In Extended Data Fig. 3d, we show the analysis of Fig. 3a (comparing the instantaneous decoder and 3g cross decoder time locked to 3g cross) zoomed out from -4 seconds to 4 seconds.

**Overlay of decoder accuracy and force**

We overlayed decoder accuracy and force in normalized units to visualize the time course of modulation. First, we centered the data; we defined one time point (“t0”) to plot at y=0 in normalized units, and we subtracted the trial-averaged data at t0 from all time points. Second, we scaled the data; we divided the centered data by the maximum trial-averaged value in a given window for normalization.

- In Fig. 2f, t0 = -4 seconds from force peak. The normalization window was from -4 seconds to 4 seconds.
- In Fig. 3a, t0 = -0.5 seconds from 3g cross. The normalization window was from -0.5 seconds to 0 seconds.
- In Extended Data Fig. 3c, t0 = -4 seconds from 3g cross. The normalization window was from -4 seconds to 4 seconds.

**Time course of SVM projection**

We visualized the time course of action-specific population activity by analyzing its projection onto the SVM dimension $w$. At each time point, the vector of population activity $x\in R^{N}$ (where each entry is one neuron’s activity) was projected onto the SVM dimension: $w^{T}x\in R^{1}$, resulting in a scalar. This is a weighted sum across neurons.

- In Fig. 2j, we used the SVM trained at force peak, and projected activity from pre-isolated 3g cross actions time locked to force peak.
- In Fig. 3ef, we used four different SVMs: the “early preparation SVM” trained at ~133ms (4 imaging frames) before 3g cross, the “late preparation SVM” trained at ~67ms (2 imaging frames) before 3g cross, the “3g cross” SVM trained at 3g cross, and the “force peak SVM” trained at force peak. We projected activity from pre-isolated 3g cross actions time locked to force peak onto each of these four SVM dimensions.
- In Fig. 3g, we plotted the “early preparation SVM” projection on the x-axis and “force peak SVM” projection on the y-axis for pre-isolated 3g cross actions.
- In Fig. 5h,i,n, we used the SVM trained at force peak, and projected activity locked to holographic stimulation.

**Time course of SVM-weighted neural ensembles**

We analyzed the activity of push weighted and pull weighted neural ensembles that biased the SVM to predict push or pull.

- We averaged the activity over the neurons in the push weighted and pull weighted ensembles time locked to force peak of 3g cross actions. (Fig. 2kl).
- We separately analyzed the average activity of the identified D1- and D2-MSNs within these ensembles (Fig. 2mn).
- In Fig. 3h-m, we defined the push weighted and pull weighted ensemble for each of four SVM’s (early preparation, late preparation, 3g cross, and force peak SVMs).
- In Fig. 5k, we averaged ensemble activity in the 100ms before force peak of 3g cross actions.
- In Fig. 5lm, we time locked ensemble activity to holographic stimulation.

**Holographic optogenetics experiments**

We first tested the spatiotemporal resolution of stimulation targeted to an individual striatal neuron through the GRIN lens. In Fig. 5bc, for each stimulation event, we delivered a train of 4 stimulations (3mW, 10 spirals, 10ms duration, 300ms interval between stimulations) to a targeted neuron.

We performed holographic optogenetics experiments to test the effect of stimulating specific ensembles of neurons on self-paced forelimb actions. Experiments relied on pairs of sessions in which we imaged the same field of view and neurons (see “Protocol to image the same field of view and neurons across sessions” above). On the first session, we performed 2-photon imaging of a field of view as mice performed the adaptive isometric task, used Suite2p to extract functional ROIs (see “Neuron detection and activity extraction with Suite2p” above), and automatically labelled ROIs as D1- or D2-MSNs based on expression of tdTomato (see “Distinguishing D1- and D2-MSNs” above). Then on the second session, we performed the 2-photon stimulation experiment consisting of a calibration block and a stimulation block (Fig. 5f). For each stimulation session, we chose to stimulate either D1- or D2-MSNs based on balancing the number of sessions for each cell type and based on the number of potential neurons to stimulate for a given session.

Within the calibration block, we performed 2p imaging as mice performed the adaptive isometric task for up to 30 minutes. We used a microscope software feature (the “Brightness over Time” feature in Bruker’s “PrairieView” software) to pre-define ROIs identified from the previous session and rapidly extract their activity. Concretely, we defined a circle for each ROI with center and radius based on each Suite2p mask, and extracted fluorescence averaged over the pixels within each circle. In addition, we extracted the behavior data (see “Data extraction and synchronization” above) and identified 3g cross actions. We took the ROIs for either D1- or D2-MSNs, z-scored each ROI’s fluorescence across the recording and fit an SVM (see “Support Vector Machine (SVM) decoder model” above) to predict push versus pull using single trials of neural activity averaged in the 5 imaging frames centered at force peak. If SVM accuracy on test trials was above 55%, we used the SVM weights to identify a push weighted ensemble and pull weighted ensemble with equal numbers of neurons (see “SVM-weighted neural ensembles” above). We then plotted the activity averaged over neurons in each ensemble time locked to force peak of 3g cross actions. If the push (pull) ensemble activity was greater for push (pull), we proceeded to perform holographic stimulation.

We defined a software command (using the “Prairielink” API to the 2p microscope) to specify holographic stimulation for each ensemble:

- Holographic stimulation was targeted to the centers of each ROI in the ensemble
- The total power was calculated as the product of the number of neurons per ensemble and a desired average power per neuron. The average power per neuron was 3-7mW, decided based on stimulation tests before the calibration block.
- Stimulation duration was typically 100ms, as this was sufficient to elicit a response in neurons expressing ChRmine.
- In order for stimulation to paint each ROI’s circular extent, dedicated galvos traced 5 spirals per stimulation with a diameter matching the average diameter of ROIs.
- We specified that this stimulation would be delivered upon a trigger signal (a force threshold crossing).

Within the stimulation block, we performed 2p imaging and closed-loop holographic stimulation as mice performed the adaptive isometric task for 30 minutes. We pre-defined a schedule that interleaved trials for what actions triggered stimulation (3g cross push and pull), and what stimulation pattern to deliver (push weighted ensemble, pull weighted ensemble, and no stimulation). Explicitly, we alternated between blocks of 6 stimulation trials triggered by 3g cross push and triggered by 3g cross pull. The stimulation patterns delivered in each trial of the block were: 1. No stimulation, 2. Push ensemble, 3. No stimulation, 4. No stimulation, 5. Pull ensemble, 6. No stimulation. We imposed a 2 second inter-trial interval between these self-paced trials.

Our custom setup (Fig. 5d, Extended Data Fig. 7b) enabled us to choose what force threshold crossing (3g cross push or pull) served as a trigger in interleaved trials and to trigger stimulation in short (~2ms) latency from the force threshold crossing, which was critical as force changed rapidly on the order of ms. The microscope computer ran custom Matlab code to step through these scheduled trials, sending software commands for stimulation through the “Prairielink” API and using a NIDAQ (USB-6001) for digital inputs and outputs to select and enable force thresholds for triggering stimulation. It sent a digital signal to a MUX to select what force threshold (3g cross push or pull) was routed to the microscope as a trigger signal. It also sent a digital signal to an in-series AND gate to enable the trigger to reach the microscope; this ensured stimulation did not occur in inter-trial intervals. It received the trigger signal (the output of the MUX) in order to track when stimulation was delivered and advance to the inter-trial interval and next trial.

**Effect of holographic stimulation on actions**

We compared force in stimulation trials versus no-stimulation trials that immediately preceded stimulation trials. No-stimulation trials that occurred closer in time to the previous stimulation trial than the next stimulation trial were excluded. We analyzed force sampled at 1 kHz and low pass filtered at 30 Hz (10^th^ order Butterworth filter with 30 Hz cutoff frequency and second-order sections output, “scipy.signal.butter” in Python), providing both high resolution visualization and de-noising.

Given that stimulation occurred with a random (but small) latency from 3g cross, we performed an alignment procedure to match the force of no stimulation trials to stimulation trials in the time lead up to stimulation. We identified the time in the no-stimulation trial that best matched the following stimulation trial on 1) the force at time of stimulation (“target instantaneous force”) and 2) the average force in the 100 ms window preceding time of stimulation (“target preceding force”). Concretely, we identified the time in each no-stimulation trial that minimized a cost, which was the sum of two terms: 1) the absolute difference between force at the time in the no stimulation trial and the “target instantaneous force”, and 2) the absolute difference between the average force in the 100ms preceding the time in the no-stimulation trial and the “target preceding force.” This alignment was performed on the raw 1 kHz force trace before filtering at 30 Hz.

We analyzed the difference between stimulation trials locked to stimulation time and no stimulation trials locked to this aligned time. We analyzed conditions pooled into “congruent stimulation” and “non-congruent stimulation,” each of which contained two conditions (Fig. 5f). Congruent stimulation consisted of 1) stimulation of the push ensemble triggered on push 3g cross, and 2) stimulation of the pull ensemble triggered on pull 3g cross. Non-congruent stimulation consisted of 1) stimulation of the push ensemble triggered on pull 3g cross, and 2) stimulation of the pull ensemble triggered on push 3g cross. Accordingly, the number of data points for statistics (Fig. 6ef) was $2*num\_sessions$, the number of pooled conditions across sessions.

**Statistical analysis**

Statistical information in Extended Data Table 1 and 2.

1. Sommer, C., Straehle, C., Köthe, U. & Hamprecht, F. A. Ilastik: Interactive learning and segmentation toolkit. in *2011 IEEE International Symposium on Biomedical Imaging: From Nano to Macro* 230–233 (2011). doi:10.1109/ISBI.2011.5872394.
